# Cyclic Peptides as Modulators of Protein–Protein Interactions: A Survival Guide from Discovery Platforms to AI-Driven Design

**DOI:** 10.3390/ijms27136067

**Published:** 2026-07-06

**Authors:** Sara Salvi, Pasquale Linciano, Simona Collina, Giacomo Rossino

**Affiliations:** Department of Drug Sciences, University of Pavia, Via Taramelli 12, 27100 Pavia, Italy; sara.salvi01@universitadipavia.it (S.S.); pasquale.linciano@unipv.it (P.L.); simona.collina@unipv.it (S.C.)

**Keywords:** protein–protein interactions, cyclic peptides, cyclotides, stapled peptides, stabilized α-helical peptides, nucleotide-encoded mass library screening, phage display, SICLOPPS, mRNA display, computational methods

## Abstract

Protein–protein interactions (PPIs) represent a vast and largely underexplored landscape of therapeutic targets, yet their structural features—including large, flat, and dynamic interfaces—have historically limited their druggability. In this context, cyclic peptides have emerged as a powerful class of PPI modulators, sitting at the interface between biologics and small molecules, and thus garnering key advantages of both classes. Their conformational constraint enhances binding affinity, proteolytic stability and, in some instances, cell permeability, thus enabling access to intracellular targets. This review provides an updated overview of cyclic peptides as modulators of PPIs, focusing on both conceptual foundations and practical strategies for their discovery and optimization. The main discovery approaches include natural sources, de novo design based on secondary structure mimetics, high-throughput screening, and computational approaches. Integration of these complementary strategies is crucial to enhance success rates in the discovery of effective and developable cyclic peptides. Accordingly, the present review aims to provide a practical guide for researchers entering this rapidly growing field, outlining current opportunities, methodological advances, and remaining challenges in the development of cyclic peptide-based PPI modulators.

## 1. Introduction

Protein–protein interactions (PPIs) play crucial roles in a wide range of cellular and biological processes, as they are essential to sustain long-range interactions in the crowded cellular environment. In fact, many fundamental cellular functions, including DNA replication, transcription, translation, and transmembrane signal transduction, rely on the dynamic assembly of protein complexes. Similarly, higher-order biological activities such as intercellular communication, cell-to-cell signaling, metabolic regulation, developmental control, and programmed cell death are orchestrated through finely regulated PPIs. The human interactome is estimated to comprise up to 650,000 PPIs, representing an enormous reservoir of potential therapeutic targets, since the dysregulation of many of these interactions has been linked to numerous human diseases, including cancer, infectious pathologies, and neurodegenerative disorders [1,2,3,4].

However, PPIs have only recently gained considerable attention as attractive therapeutic targets, given that traditional drug discovery has primarily focused on enzymes, ion channels, and receptors. Over the past decade, significant progress has been made in the development of PPI modulators, with several candidates advancing to clinical trials and some already approved for clinical use [5,6]. Among the most notable examples are monoclonal antibodies targeting the PD-1/PD-L1 interaction, which plays a key role in tumor immune evasion. By disrupting this interaction, these agents restore immune system activity against cancer cells [7]. From 2021, five monoclonal antibodies with this mechanism of action were approved: Dostarlimab [8], Retifanlimab [9], Toripalimab [10], Tislelizumab [11,12], and Cosibelimab [13]. In addition, a number of small-molecule PPI modulators have also reached the market. These include Fostemsavir (approved in 2021) [14], which inhibits the gp120-CD4 interaction for HIV treatment [15], and the anticancer agent Venetoclax [16], a BH3 mimetic that blocks the interaction between BCL-2 and pro-apoptotic proteins (e.g., BIM, BAX) [17]. Furthermore, alternative modalities such as stapled peptides are currently being explored as PPI modulators in both preclinical and clinical settings. A remarkable example is sulanemadlin (ALRN-6924), a stabilized α-helical peptide that inhibits p53/MDM2 and p53/MDMX interactions, currently under evaluation in phase 1b/2 clinical trials for cancer treatment [18].

Despite these advances, the structural features of PPIs remain a major challenge for drug design. PPI interfaces are typically large, flat, predominantly hydrophobic, often transient, flexible and/or dynamic. All these characteristics have historically led to their classification as “undruggable” targets. Small molecules often display limited efficacy against PPIs due to the lack of well-defined binding pockets. Moreover, attempts to extend small molecules to engage larger interaction surfaces frequently result in unfavorable pharmacokinetic properties. Monoclonal antibodies, on the other hand, offer high potency and specificity but are largely confined to extracellular targets because of their high molecular weight, limited cell permeability, and potential immunogenicity [5,19].

In this context, peptides have emerged as promising PPI modulators, as they can effectively mimic protein secondary structure elements or interaction domains, enabling selective modulation of PPIs with high affinity and specificity. Consequently, peptides have gained increasing recognition as a distinct and versatile class of therapeutic agents. However, linear peptides composed of natural amino acids often suffer from poor biophysical properties, including low proteolytic stability, limited cell permeability, and short in vivo half-lives, which hamper their clinical translation [6,20,21,22,23].

To address these limitations, chemical modifications such as macrocyclization, stapling, and insertion of unnatural or non-proteinogenic amino acids (npAAs) have been widely employed to stabilize bioactive conformations and enhance metabolic resistance [6,24,25], whereas the insertion of warheads has been explored to obtain covalent inhibitors with improved potency [26]. However, despite the effectiveness of such strategies, the rational design of effective PPI modulators remains challenging due to the intrinsic complexity of PPI interfaces and the absence of universally applicable design principles, leaving many disease-related PPIs insufficiently explored as therapeutic targets.

In the present review, we provide an updated overview of the use of cyclic peptides to target PPIs, covering key developments in the field, with a special focus over the past 10 years. Firstly, the rationale for cyclization will be described, and then we will discuss different approaches to the identification of cyclic peptides as PPI modulators. These include discovery from natural products, de novo design, display technologies, combinatorial approaches, and computational tools. As this review addresses a rapidly growing and evolving field, it does not have the ambition to be exhaustive, but rather to provide support to novices embarking on the exploration of this vast land. For this reason, we refer the reader to several recent excellent reviews and perspectives for additional information and more in-depth discussions of the topics covered herein [25,27,28,29,30,31,32,33,34].

## 2. Cyclic Peptides: Advantages and Cyclization Strategies

Since PPIs are mediated by specific amino acid residues located on the surface of interacting proteins, these epitopes can be used for the design of peptide-based inhibitors that faithfully reproduce the structural features of protein interfaces. The possibility to construct relatively large molecules by increasing peptide length is crucial to target the typically extended PPI interfaces (1500–3000 Å^2^), while the intrinsic flexibility of the peptide backbone enables better adaptation to the dynamic nature of PPIs. Therefore, owing to their physicochemical properties, conceptual simplicity and utility as scaffolds for drug design, linear peptides are useful starting points for further optimization aimed at overcoming their intrinsic limitations, including poor stability and limited cell membrane permeability. In this context, they provide conceptual building blocks for the development of improved derivatives and peptidomimetics as valuable therapeutic agents [24,27,28,29].

Various chemical modifications, such as N-terminal or C-terminal modifications, amide bond variation, incorporation of unnatural amino acids, and cyclization, can be employed to improve the chemical and biological properties of native peptides. Among these strategies, peptide cyclization has emerged as a particularly promising approach. By introducing a macrocyclic constraint, cyclic peptides are locked into their bioactive conformations, thereby enhancing their metabolic stability, bioavailability, and selectivity, compared with their linear counterparts. In detail, the conformational restriction improves binding affinity by reducing the entropic penalty associated with target engagement and enhances resistance to proteolytic degradation thanks to the lack of free N- and C-termini, as well as to the limited conformational flexibility and increased steric hindrance that reduce accessibility to the protease catalytic machinery [25,30,31]. Furthermore, cyclic peptides display superior cell permeability, enabling them to access intracellular targets, including challenging PPIs. According to the ‘‘chameleon hypothesis’’, cyclic peptides exhibiting smart conformational dynamics (shielding or exposing their polar functionalities in response to the environment) possess enhanced cell membrane permeability due to their transiently reduced surface polarity and area [30,32,33,34].

Due to this unique combination of advantages, cyclic peptides garnered considerable attention from the scientific community in recent years and have been applied in different fields beyond PPI modulation, including the design of enzyme agonists and antagonists, imaging and diagnostic tools, modulators of protein–RNA interaction, and innovative materials for biomedical applications [35,36,37,38,39,40,41]. To date, more than fifty cyclic peptides have reached regulatory approval for different applications (either therapeutic or diagnostic), as indicated by PepTherDia [42] and Drug Bank [43]. Most of them are derived from natural products and are used as anti-infective and anticancer agents.

**Figure 1:**
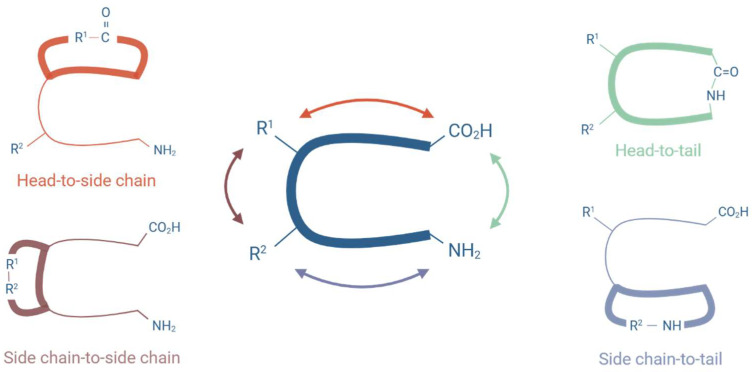
Peptide cyclization strategies.

From a synthetic standpoint, peptide cyclization can be achieved through four principal modes: head-to-tail (C-terminus to N-terminus), head-to-side chain, side chain-to-tail, and side chain-to-side chain (Figure 1). The choice of cyclization strategy is not merely dictated by synthetic feasibility but is closely related to the desired structural and functional properties of the resulting peptide. Different cyclization topologies can impose distinct conformational constraints, thereby influencing secondary structure stabilization, binding affinity, and ADME profile. For instance, side chain-to-side chain cyclization can be exploited to generate stapled peptides that stabilize α-helical conformations. While numerous reviews have comprehensively covered the synthetic methodologies available [44,45,46,47,48], an increasing body of evidence highlights the role of cyclization as a key design element. Such influences will be better elucidated in the following chapters.

## 3. Discovery of Cyclic Peptides as PPI Inhibitors

A wide variety of strategies have been developed to identify cyclic peptides as inhibitors of PPIs. From nature-aided approaches to de novo design, including high-throughput screening and artificial intelligence (AI)-driven techniques, the following sections provide an overview of these methods, acknowledging that their effective application often relies on the combination of multiple approaches.

### 3.1. Natural Sources

Nature displays remarkable structural diversity in the realm of cyclic peptides. Many of these molecules exhibit potent biological activities, and more than 40 naturally occurring cyclic peptides or their derivatives are currently used as therapeutic agents [29,31,49,50].

Despite the recent surge in AI- and computationally driven strategies for the design of novel cyclic peptides, natural sources still represent the predominant origin of these compounds. Indeed, according to the CyclicPepedia database, which currently reports 8751 known cyclic peptides, 62.50% of them derive from natural sources, including animals (57.97%), plants and fungi (32.08%), and bacteria (10.43%) [51].

The discovery of natural peptides can serve as a valuable source of inspiration for the development of novel cyclic peptides with improved biological activity and pharmacokinetic properties. In this context, cyclotides represent one of the most notable examples. They are defined as plant-derived macrocyclic peptides, characterized by the presence of a structural motif known as the cyclic cystine knot (CCK), which is responsible for their great structural stability and conformational rigidity [52,53]. This unique architecture enables cyclotides to function as robust scaffolds, capable of tolerating sequence insertions and molecular evolution approaches, thereby facilitating the development of cyclic peptides involved in PPI inhibition. The cyclotides MCoT-I and MCoT-II are among the most widely used scaffolds for molecular grafting, enabling the incorporation of bioactive peptide epitopes with otherwise limited stability and bioavailability, while preserving their biological activity [49]. Philippe et al. recently exploited this strategy to develop a stabilized p53:MDM2/MDMX inhibitor as an anticancer agent with increased cellular uptake [54]. The field has expanded considerably in recent years, with substantial efforts devoted to overcoming the synthetic limitations associated with cyclotide grafting. In particular, innovative modular strategies, such as the recently reported “plug-and-play” approach, enable the insertion of complex bioactive epitopes into pre-folded cyclotide scaffolds, thereby bypassing folding bottlenecks and significantly expanding the scope of molecular grafting [55].

#### 3.1.1. Bioassay-Guided Fractionation

The most consolidated strategy for the discovery of novel natural products is bioassay-guided fractionation. Extracts from plant matrices, animal tissues, or microorganism cultures are obtained using solvents of increasing polarity. The crudes are then screened for the desired biological activity, and the most active samples undergo additional rounds of fractionation to isolate the active compound [56]. The final purified compound is characterized using analytical techniques such as high-performance liquid chromatography (HPLC), nuclear magnetic resonance (NMR), and mass spectrometry (MS). One of the most relevant examples in this context is chlorofusin, a cyclic peptide isolated via bioassay-guided screening of microbial extracts from *Microdochium caespitosum*, which disrupts the p53/MDM2 interaction [57]. This approach is also compatible with high-throughput screening (HTS) formats, enabling the parallel evaluation of large extract collections followed by iterative fractionation of confirmed active samples. Despite its historical success, activity-guided fractionation is hampered by several limitations. The process is biased toward abundant constituents, and activity can be lost during fractionation due to synergistic effects, chemical degradation, or irreversible binding to chromatographic stationary phase. Furthermore, the iterative nature of the workflow makes it time-consuming and costly, and frequently leads to the rediscovery of already known compounds. These constraints have motivated the development of complementary sequence-guided strategies, as discussed in the following section [58,59,60].

#### 3.1.2. Sequence-Guided Discovery

Over the past two decades, the increasing availability of high-quality genomes and de novo transcriptomes have enabled a shift from traditional bioassay-guided fractionation to sequence-guided discovery. In this approach, genes encoding peptide precursors and their maturation enzymes are identified through in silico mining, enabling direct prediction of peptide structures and biosynthetic logic. This genomics-driven strategy provides rapid and systematic access to natural peptide diversity while bypassing many of the constraints associated with purification from complex extracts, as already mentioned above.

A growing body of reviews and research articles now outlines how genomic, transcriptomic, and peptidomic data can be integrated to identify novel cyclic peptides across plants, animals, and microorganisms, highlighting sequence-guided discovery as a central route for future natural product exploration [61,62]. For instance, the genome mining-driven expansion of the lanthipeptide family, a class of polycyclic peptides characterized by intramolecular thioether bridges, has provided a rich source of structural scaffolds for biotechnological applications. Notably, the genome mining-guided discovery of the highly substrate-tolerant synthetase ProcM in the marine cyanobacterium *Prochlorococcus* enabled the development of large genetically encoded lanthipeptide libraries, from which inhibitors of specific PPIs have been identified [63,64].

### 3.2. De Novo Design

While natural cyclic peptides have provided valuable scaffolds for modulating PPIs, their structural complexity and limited availability often restrict their direct use as drug candidates. De novo design strategies have therefore emerged as a complementary approach, enabling the development of novel cyclic peptides, even without prior structural templates. This approach allows the identification of new structures with tailored physicochemical and biological properties, while probing unexplored chemical space.

In particular, structure-based design of PPI inhibitors requires a detailed understanding of the interaction interface. Ideally, the three-dimensional architecture of the complex is experimentally determined at high resolution. Nevertheless, remarkable advances in computational modeling and, more recently, in AI-driven structure prediction have substantially expanded the availability and reliability of protein complex models. These developments have significantly broadened the applicability of structure-based approaches, as will be discussed in Section 3.4.

Once the structure of the interaction interface is available, the so-called “hotspot residues” can be identified to pinpoint the amino acids that contribute most significantly to binding affinity and are therefore essential for maintaining the interaction. Specifically, hot spots are defined as key residues whose mutations significantly reduce binding affinity (ΔΔG ≥ 2 kcal/mol) and form cooperative regions that involve specific residue combinations and unique surface arrangements. Hot spots function through tightly packed, networked interactions, providing flexibility and the ability to bind multiple partners. The identification of hotspots can be achieved through experimental techniques, such as site-directed mutagenesis (i.e., alanine scanning), or through computational approaches, which over the past decade have proven to be more cost-effective and less time-consuming [65,66].

The secondary structure adopted by the peptide-binding epitope can be mimicked to generate a cyclic peptide that preserves the spatial orientation of key side chains. The specific secondary motif to be reproduced dictates the most appropriate stabilization strategy to achieve the desired conformational constraint and bioactive geometry [67].

#### 3.2.1. Stabilized α-Helical (SAH) Peptides

α-helices represent the predominant structural motif within PPI interfaces, with residues from helical segments participating in approximately 62% of all protein–protein interactions [68]. Stabilized α-helical peptides, particularly stapled peptides, have therefore emerged as a major class of PPI inhibitors. The term stapling refers to the introduction of an external covalent constraint that enforces and stabilizes a specific secondary structure, typically an α-helix [69]. This strategy enhances membrane permeability and proteolytic stability while maintaining the bioactive geometry required for target binding.

**Figure 2:**
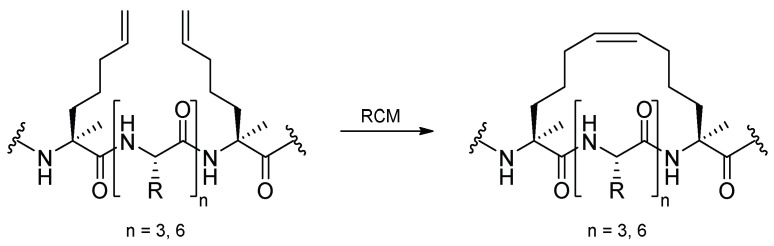
General structure of an all-hydrocarbon stapled peptide obtained through RCM.

When designing an SAH peptide, the first parameter to consider is the position of the residues to be crosslinked. In α-helices, side chains are spatially aligned at positions i, i + 4, i + 7, and i + 11, with i, i + 4 and i, i + 7 representing the most frequently employed spacing patterns. Once the linkable residues are defined, the related side chains are connected through an appropriate stapling strategy. Several reviews provide in-depth discussions of this topic, including the comprehensive work by Li et al., which systematically outlines the available chemical approaches for crosslinking two anchoring residues [70]. Among these, the most widely used technique is all-hydrocarbon stapling, in which the covalent bridge consists of a hydrocarbon chain typically formed through a ruthenium-catalyzed ring-closing metathesis (RCM), as schematized in Figure 2. This method requires the incorporation of two α-α disubstituted non-natural amino acids bearing terminal alkenes at the appropriate positions within the peptide sequence, ensuring the reactive groups are correctly oriented to undergo efficient cyclization [71,72]. This approach was used in the development of the aforementioned compound ALRN-6924, which binds to both MDM2 and MDMX by mimicking the N-terminal domain of the p53 tumor suppressor protein [18].

**Figure 3:**
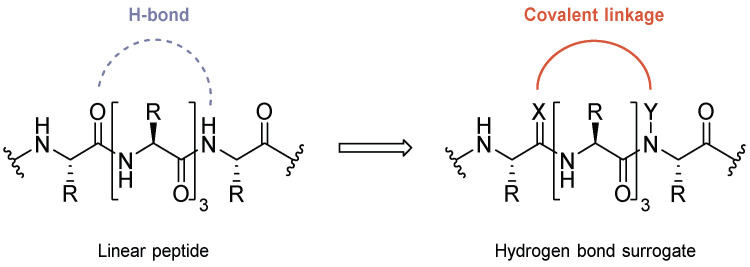
General structure of a hydrogen bond surrogate.

An additional class of stabilized α-helical peptides is represented by hydrogen bond surrogates (HBSs). In this approach, the i, i + 4 hydrogen bond on the peptide backbone is replaced with a stable covalent linkage to enforce a conformationally stable helix (Figure 3). HBSs differ from stapled peptides because the crosslink connects the peptide backbone rather than the side chains. The result is a reinforcement of intramolecular hydrogen bonds, providing a nucleation site that promotes helix initiation and stabilizes the overall helical conformation [73].

Several HBS α-helices have been developed specifically to disrupt PPIs, demonstrating the versatility of this strategy in targeting biologically relevant interfaces that are often considered challenging for conventional small molecules. Representative examples include pro-apoptotic mimetics designed to target the Bak/Bcl-x_L_ interaction, where stabilization of the α-helical BH3 domain enhances binding affinity toward Bcl-2 family proteins [74,75]. HBS technology has also been applied to the development of antimalarial peptides that inhibit the formation of the MyoA/MTIP complex, a key component of the glideosome machinery required for parasite motility and host–cell invasion [76]. In oncology, HBS peptides have been engineered to disrupt the aforementioned p53/MDM2 interaction, thereby restoring p53 tumor suppressor activity in cancer cells [77].

#### 3.2.2. Mimetics of β Secondary Structure

**Figure 4:**
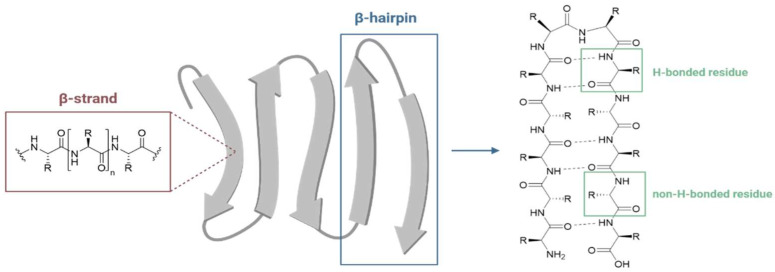
General structure of an antiparallel β-sheet.

β-sheets are the most abundant secondary structure in proteins after α-helices. They are composed of β-strands, which constitute the structural units of this motif. A β-strand is defined as a polypeptide chain in an extended conformation, where alternating side chains are oriented in opposite directions. Multiple β-strands aligned through interstrand hydrogen bonds assemble into a β-sheet, which can be classified either as parallel or antiparallel depending on the relative orientation of the strands. When they run in the same direction, the sheet is referred to as parallel; conversely, opposite orientations define an antiparallel β-sheet [78]. Within antiparallel β-sheets, the β-hairpin motif can be recognized, consisting of two antiparallel β-strands connected by a loop or a turn, typically involving two residues, as outlined in Figure 4. The amino acid residues along each strand adopt a zigzag arrangement, with side chains alternately oriented on opposite sides of the β-strand. Accordingly, residues are classified as H-bonded and non-H-bonded, the former being capable of forming interstrand C=O–HN hydrogen bonds with the aligned residue on the opposing strand.

Among the aforementioned structural motifs, β-hairpins exhibit the most suitable geometry for designing cyclic peptides. Several examples of cyclic β-hairpin mimetics have been reported and will be discussed in the following section, followed by β-strands and β-sheet mimetics, which are less common yet synthetically accessible.

#### 3.2.3. β-Hairpin Mimetics

**Figure 5:**
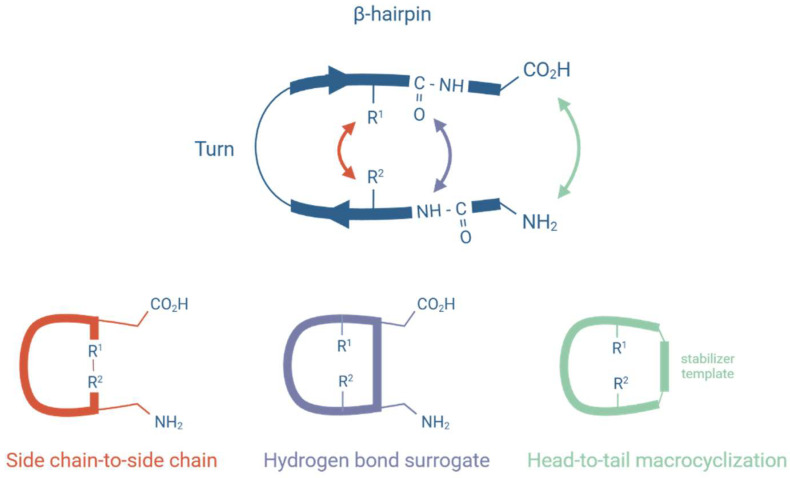
β-Hairpin stabilization techniques.

Several strategies have been developed to stabilize the hairpin conformation, which can be classified into the three main categories reported in Figure 5: head-to-tail macrocyclization, side chain stapling, and hydrogen bond surrogate (HBS).

Head-to-tail macrocyclization aims to connect the terminal amino acids of the antiparallel strands to form a conformationally constrained cycle. The most extensively studied approach involves the introduction of a “hairpin stabilizer template”, typically a d-Pro-l-Pro dipeptide, which adopts a rigid type-II’ β-turn conformation [79,80]. This motif effectively promotes β-hairpin folding and facilitates the formation of cross-strand hydrogen bonds, providing a nucleation site at the first residue pair attached to the template.

The β-hairpin can also be stabilized through the formation of side chain-to-side chain interstrand bridges, which may be either covalent (e.g., disulfide or 1,2,3-triazole ring) or noncovalent (such as π–π interaction or cation–π interaction) [67]. β-strand-enforcing amino acids also belong to this category: rigid building blocks are incorporated into the peptide sequence to reproduce the hydrogen-bonding pattern of β-hairpins. In some cases, it is possible to establish a covalent linkage between two β-strand-enforcing residues positioned on opposite strands [81].

**Figure 6:**
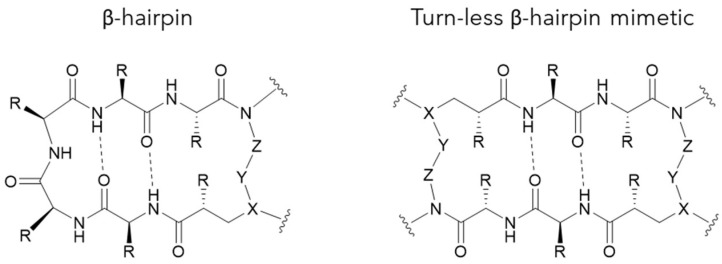
General structure of β-hairpin and turn-less β-hairpin mimetic.

The HBS strategy, as previously described in Section 3.2.1, differs from side chain stapling since the antiparallel β-strands are covalently connected through the peptide backbone, effectively replacing the interstrand hydrogen bond normally formed between opposing C=O and NH groups. Although originally developed to stabilize α-helices, this approach has also been successfully applied to β-hairpin stabilization by Sawyer et al., who identified thioether and disulfide bridges as ideal HBS linkers for achieving both conformational stabilization and protease resistance [82]. More recently, an alternative β-hairpin mimetic, named turn-less antiparallel β-hairpin mimic, has been reported (Figure 6). In this approach, the conventional β-turn is replaced by an additional HBS linkage, through which the two β-strands are directly connected via backbone covalent constraints, resulting in a macrocyclic architecture that reproduces the spatial arrangement of a β-hairpin without relying on a turn motif (Figure 6). This strategy eliminates the contribution of the β-turn to motif stabilization, which often depends on the specific strand sequence [83].

When stabilizing a β-hairpin, a key aspect to consider is the hydrogen-bonding pattern of the native motif being mimicked. Indeed, the introduction of additional residues, crosslinking bridges, or turn-inducing elements can alter the alignment of the H-bonded pairs, which must remain properly matched to maximize interstrand hydrogen bonding and stabilization of the hairpin. Therefore, the position of hydrogen-bonded residues should be carefully identified and taken into account during the design of the mimetic.

#### 3.2.4. β-Strand Mimetics

Isolated β-strands play a significant role as protein-binding epitopes (PBEs) in PPIs, with proteases representing some of the most frequently targeted systems. To mimic β-strand motifs, the most commonly employed cyclization strategies involve side chain-to-side chain or side chain-to-main chain linkages, typically connecting residues at the i, i + 2 positions, which is optimal for preserving the characteristic geometry of β-strands and the alternating orientation of side chains [84].

**Figure 7:**
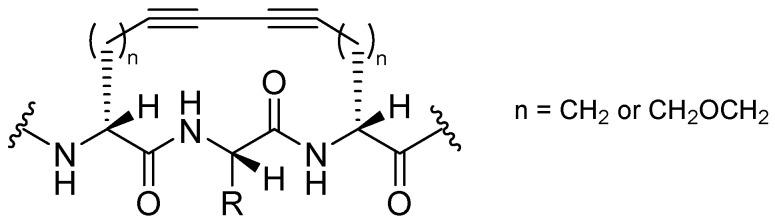
General structure of β-strand mimics with rigid diyne brace.

More recently, innovative cyclic β-strand mimics have been reported by Adams et al., who introduced a rigid diyne brace between side chains in positions i, i + 2 of short peptides (Figure 7) [85]. This strategy yields conformationally constrained mimetics with an extended backbone, enabling a more faithful reproduction of native β-strand geometry and resulting in improved binding affinity and selectivity.

#### 3.2.5. β-Sheet Mimetics

Since the distinction between β-sheet, β-hairpin, and β-strand mimetics is not always clearly defined in the literature, we refer here to β-sheet mimetics as those peptidomimetics displaying a higher level of structural complexity compared to β-hairpin mimetics. In this context, the rational design of cyclic peptides capable of faithfully reproducing β-sheet architectures remains particularly challenging. As discussed above, the β-strands composing a sheet must be precisely aligned to maximize the long-range hydrogen-bonding network that stabilizes this secondary structure. In addition, β-sheets are highly prone to aggregation and misfolding when removed from their native protein context.

To address these challenges, more advanced design strategies have been developed to stabilize higher-order β-sheet mimetics by incorporating multiple strands within a single cyclic or multicyclic framework. Among these, one of the most effective approaches involves the use of bicyclic or multicyclic peptides, in which distinct cyclization events are employed to simultaneously stabilize individual β-strands and enforce their relative spatial orientation [86,87].

Despite these advances, β-sheet mimetics remain less common than α-helical or β-hairpin-based inhibitors, primarily due to synthetic complexity and persistent challenges related to solubility and conformational control. Nevertheless, when successful, cyclic β-sheet mimetics offer unique opportunities for targeting PPIs, particularly in cases where isolated β-hairpin or β-strand motifs are insufficient to recapitulate the native binding interface.

### 3.3. High-Throughput Screening (HTS)

An effective counterpart to structure-based design approaches is the identification of bioactive cyclic peptides through screening of combinatorial or random libraries. While structure-based strategies rely on prior knowledge of the target and its binding interface to rationally optimize peptide topology and conformation, library HTSs empirically explore structural diversity, enabling the discovery of functional hits even in the absence of detailed structural information. Such methods leverage large collections of cyclic peptides, either synthetically generated or biologically expressed, to identify binders with favorable affinity, stability, and membrane permeability profiles.

Given the size and structural complexity of these libraries, chemical synthesis is often impractical and therefore other approaches have emerged over the past decades. In particular, genetically encoded libraries take advantage of biological transcription and translation machinery to produce peptides that are linked to their encoding nucleotide sequence. This approach, often referred to as nucleotide-encoded mass library screening (NELS), allows an easier production of large libraries (hundreds of millions of molecules) leveraging in vitro biosynthesis, while also enabling the deconvolution of potential hits thanks to the nucleotide tag. Moreover, this kind of screening is compatible with both functional and affinity-based screening, even in cells. In the following sections (Section 3.3.1, Section 3.3.2, Section 3.3.3 and Section 3.3.4), specific NELS techniques will be discussed more in detail [48,88,89].

#### 3.3.1. Phage Display

**Figure 8:**
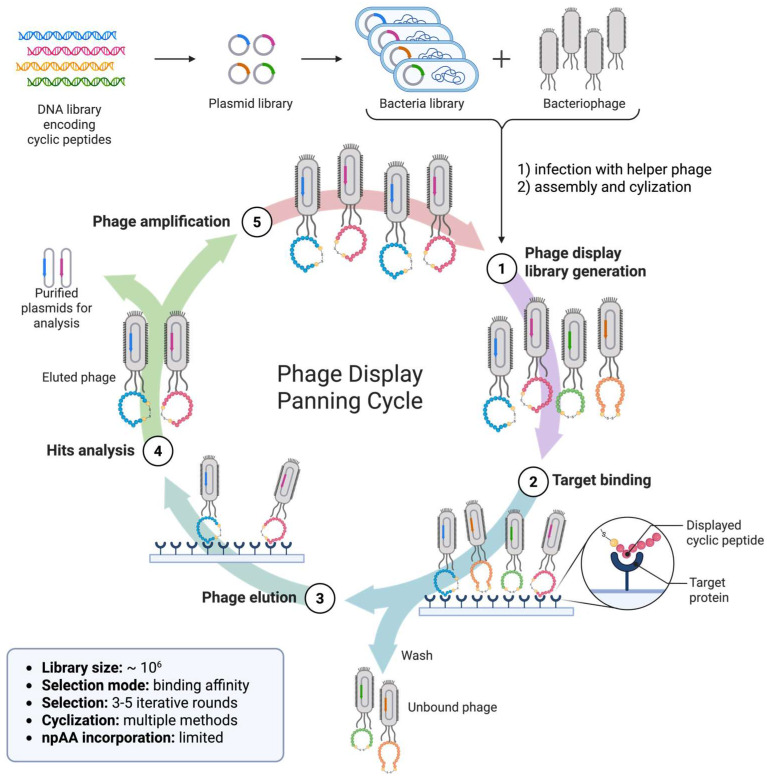
Schematization of the phage display panning cycle for the identification of cyclic peptides. The designed DNA library is initially transformed in *E. coli.* using the corresponding plasmids. Helper phages then infect the bacteria, generating a peptide library displayed on the phage coat protein. These peptides can be cyclized by different means (in the figure, disulfide bridges are used as a representative example). The phage library is then screened against an immobilized target, which captures the binders, while unbound phages are washed away. Then, the bound phages are eluted from the target substrate and the resulting hit sequences can be amplified through new rounds of selection, before ultimate sequencing.

Phage display links genotype (phage DNA) and phenotype (a displayed peptide/protein) on the surface of a bacteriophage (i.e., viruses that infect bacteria), and it constitutes one of the most popular high-throughput strategies in peptide discovery [88,89,90,91]. In detail, a randomized DNA library encoding peptide variants is inserted into a phage coat protein gene (commonly pIII or pVIII of filamentous phage M13). Upon expression in a bacterium host (e.g., *E. coli*), each phage produces and displays one peptide variant on its surface and carries inside the genome that encodes it. Since the peptide remains attached to the surface of the phage, it is possible to isolate the phages displaying desirable peptides from large collections, using the immobilized target protein as bait. Upon binding to the target, washings are performed to remove weak or non-specific binders. Then, bound phages are recovered by elution and used to infect *E. coli*, regenerating a pool enriched in binders (amplification). This process is typically repeated 3 to 5 times in an iterative cycle called panning (Figure 8), to sharpen enrichment toward high-affinity and high-specificity peptides. Finally, the DNA of the selected phages is sequenced to establish the identity of selected peptides. In this platform, cyclization typically involves the formation of disulfide bridges from cysteine residues in the linear sequence, although different cyclization strategies have been explored [90]. This approach also enables the display of multicyclic peptides, as discussed in detail by Chen et al. in their recent review on the topic [91].

Despite the high popularity of phage display, due to its relatively simple setup and cost-effectiveness, some limitations have long hampered its full potential. Most importantly, since it consists of an in vivo expression system, this technique was initially limited to the use of the 20 proteinogenic amino acids. This drawback has been addressed either by introducing non-proteinogenic amino acids (npAAs) synthetically on the cyclizing scaffold (by means of reactive handles) or by ribosome-mediated genetic code expansion, using aminoacyl-tRNA-synthetases (aaRS)/tRNA pairs orthogonal to the *E. coli* system [92]. The other historically relevant limitation of phage display is the size and structural diversity of the DNA library, typically limited to about one billion of variants. For easy targets such as proteins that bind to short, linear peptides, small libraries (10^3^–10^6^ different peptides) are sufficient. Instead, the development of cyclic peptides for challenging targets requires much larger libraries: in addition to target engagement, additional qualities such as high stability or binding to multiple targets (both human and animal homologues of a protein) are often required. Two main practical aspects limit the size of phage display libraries: the generation of DNA vectors coding for many different peptides and the transformation of vector DNA into multiple bacterial cells [88,90]. Notably, Carle et al. have recently achieved an important increase in the phage display library size, generating a 100-billion-(bi)cyclic-peptide library with high skeletal diversity. This milestone was accomplished via a combination of whole plasmid PCR with small phagemid vectors that facilitated bacterial transformation [93].

#### 3.3.2. Split Intein-Mediated Circular Ligation of Peptides and Proteins (SICLOPPS)

**Figure 9:**
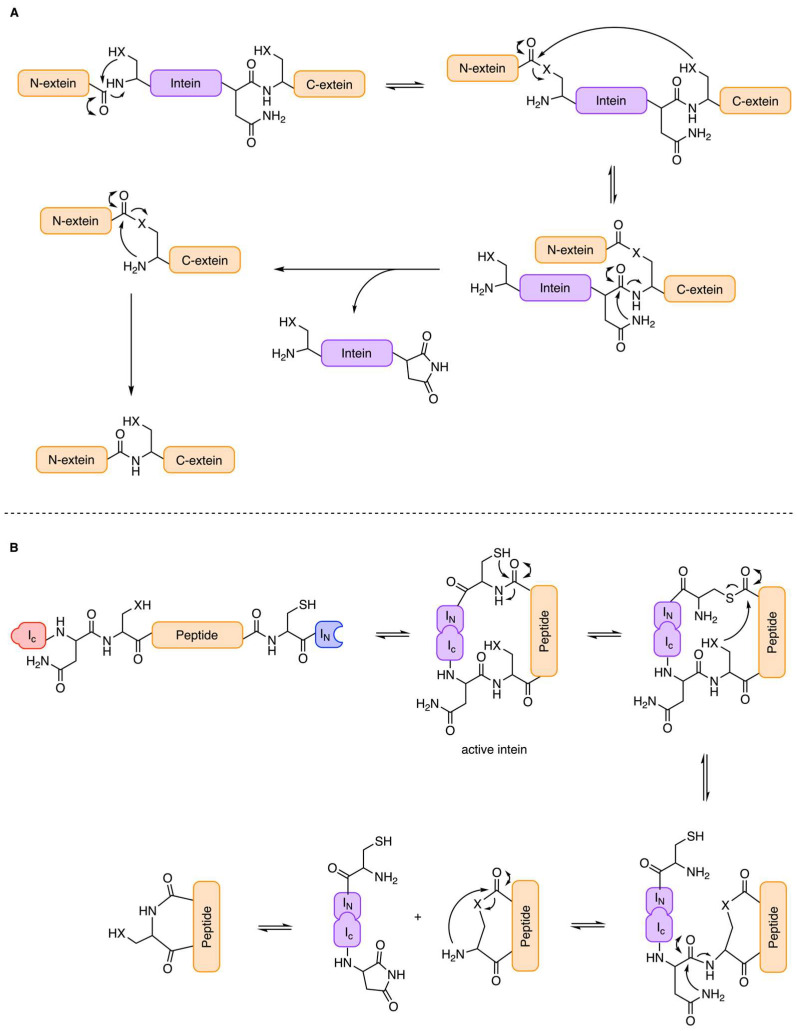
(**A**) Molecular mechanism of protein splicing: the internal portion of the protein sequence (i.e., the intein, shown as purple box) is excised while the N- and C-terminal sequences (orange boxes) are ligated to produce a contiguous polypeptide. (**B**) Molecular mechanism of SCICLOPPS: the extein constitutes the target peptide (orange box), which is flanked by the C-terminal and N-terminal segments of a split intein (IC and IN, shown as red and blue boxes respectively). Upon proper folding, the active intein is formed (purple box), which leads to the formation of the target cyclic peptide. In the schemes, XH represents a nucleophilic substituent (e.g., OH, SH).

SICLOPPS is a technology that exploits protein splicing to generate genetically encoded cyclic peptide libraries in a cell-based system. Protein splicing is a posttranslational process consisting of an autocatalytic and intramolecular reaction of a protein composed of an internal segment called intein, flanked by two external segments named exteins (N-extein and C-extein according to the terminus they contain). As depicted in Figure 9A, splicing occurs when the intein catalyzes self-excision from the precursor protein to ligate the two exteins via a peptide bond. The reaction involves an amino acid with a nucleophilic side chain (typically a cysteine or a serine) [94].

In the split intein system of SICLOPPS, schematized in Figure 9B, two complementary half-inteins (N-intein and C-intein) are placed at the two sides of a single peptide of interest that replaces exteins. Splicing occurs with a similar mechanism, but in this case bringing the two half-inteins into proximity to reconstitute the full intein produces a cyclic peptide as a byproduct [48,88,95]. A wide range of diverse peptides can be cyclized with this approach, provided that the first amino acid in the extein is a nucleophilic cysteine or serine residue.

**Figure 10:**
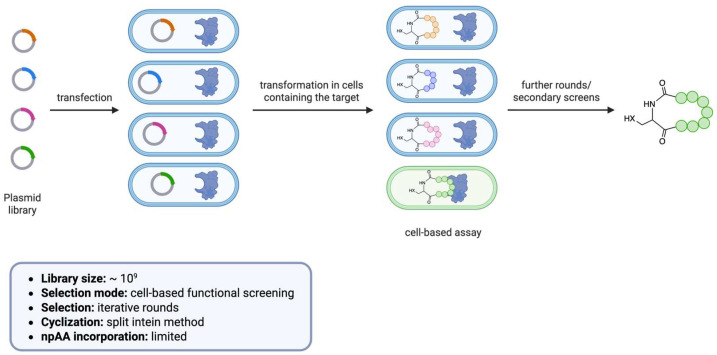
SICLOPPS is carried out in living cells, enabling the simultaneous expression of both the plasmid library and the target protein. The peptide is cyclized head-to-tail via the split intein method, while the selection of hits can be performed using a range of cell-based assays.

The number of randomized residues in the cyclic peptide library is determined by the transformation efficiency of the host organism (e.g., *E. coli*, yeasts or human B cells), which typically is up to about 6–7 amino acids, thus allowing for libraries of 10^6^–10^9^ members. Despite being mainly restricted to the use of proteinogenic amino acids, an orthogonal aaRS/tRNA pair can be used for incorporating a single npAA, similarly to phage display approaches. A key feature of SICLOPPS is its intracellular mode of selection, which enables the screening of cyclic peptides based on functional activity rather than target binding (Figure 10). This ensures the obtainment of truly bioactive compounds, selected within the complex intracellular space and exhibiting stability and efficacy in a native cellular context. Moreover, SICLOPPS is compatible with a broad range of cell-based assays (fluorescence, vitality, etc.), facilitating efficient and straightforward functional screening [48,88,95].

Recently, McDermott et al. reported on the identification of macrocyclic inhibitors of the HIF-1α/HIF-1β protein–protein interaction, thanks to a revised and updated SICLOPPS screening protocol [96]. Hypoxia-inducible factor 1 (HIF-1) is a transcription factor involved in the adaptation and survival of solid tumors to their hypoxic microenvironment and it is correlated with aggressive cancer phenotypes and poor patient prognosis. Therefore, inhibiting this transcription factor has gained increasing consensus as a viable strategy for the treatment of a variety of cancers.

Another example of application of SICLOPPS screening comes from work by Lennard et al., who developed a cyclic peptide inhibitor of p6/UEV protein–protein interaction for the treatment of HIV infections [97]. The interaction between the p6 domain of the HIV Gag protein and the UEV domain of the human TSG101 protein is crucial in a process known as budding, i.e., the mechanism by which an enveloped virus exits an infected host cell without lysis. Since budding involves several host proteins, it represents a promising opportunity for the development of antiviral agents with a PPI inhibitory mechanism of action.

#### 3.3.3. mRNA Display

**Figure 11:**
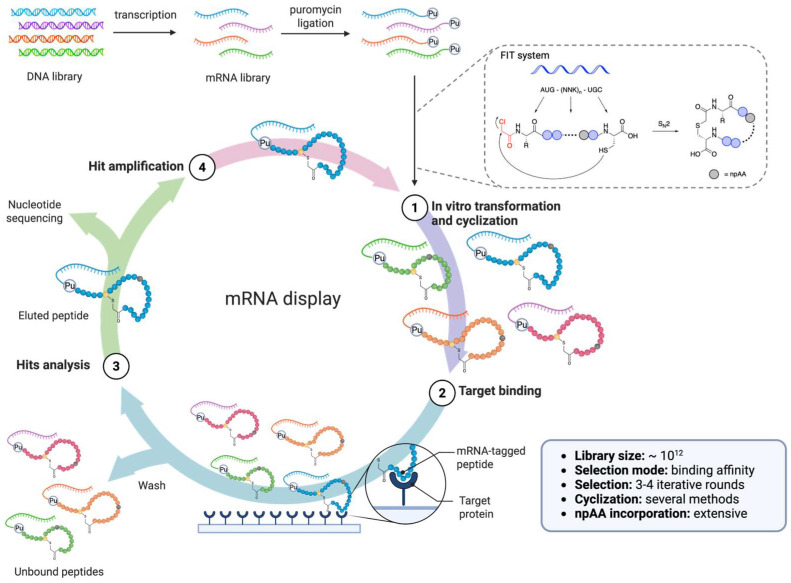
Schematization of the RaPID system, consisting of the combination of mRNA display with the flexible in vitro translation (FIT) technology. The mRNA library is initially ligated with a puromycin linker. Then, the library is translated, exploiting the FIT system to include npAAs (black spheres in the figure). The subsequent cyclization can be achieved by multiple methods (e.g., S_N_2 on a N-chloroacetyl group, as reported in the scheme). This yields mRNA-tagged cyclic peptides that are then screened against an immobilized target. The selected hits can be amplified through iterative selection cycles, prior to sequencing and identification of the hit.

mRNA display is another well-established technology for the screening of peptide libraries, and it has been successful in the identification of cyclic peptide modulators of PPIs. Notable examples include the discovery of enlicitide (MK-0616), a PCSK9 inhibitor that completed phase 3 clinical trial for hypercholesterolemia [98,99], and paluratide (LUNA18), a pan-RAS inhibitor that reached Phase 1 clinical trial for solid tumors [100,101]. In particular, mRNA offers advantages over other approaches in terms of speed and ease in the identification of peptides according to their affinity toward a target protein. Of particular relevance is the combination of mRNA display with flexible in vitro translation (FIT). This specific platform allows access to macrocyclic peptides containing non-proteinogenic amino acids (npAAs) and is known as “random nonstandard peptides integrated discovery” (RaPID) [46,47]. As outlined in Figure 11, the process starts with the in vitro transcription of a randomized synthetic DNA library (about 10^12^ members, typically encoding 6–15 amino acids sequences) to mRNA, which is conjugated to a puromycin linker, essential for covalent peptide–mRNA fusion. The FIT system is used to reprogram the genetic code in order to include npAAs (e.g., N-methyl-, D-, β-amino acids) and enable spontaneous macrocyclization, for example, by introducing a reactive N-chloroacetyl group on the N-terminal amino acid. Translation occurs in a reconstituted system, producing a cyclic peptide covalently linked to mRNA through the puromycin linker, physically connecting phenotype and genotype. Then, the tagged peptides are incubated with the immobilized target protein and those that bind with high affinity are selected, while non-binders are washed away. Subsequent amplification by PCR of the recovered genetic information produces a second mRNA library, enriched with sequences encoding the active peptides. This iterative evolution cycle is repeated (typically 4–8 times) until a pool of highly potent and selective binders is obtained. The peptide sequences are obtained by sequencing of the enriched genetic code covalently linked to the peptide ligands [102]. Different cyclization strategies are now compatible with mRNA display, from the reaction between the aforementioned N-terminal chloroacetyl function and an internal cysteine to more recent approaches enabling backbone macrocyclization [103].

The RaPID system has been recently employed by Villequey to discover peptide inhibitors for fibroblast growth factor receptor 3c (FGFR3c) [104]. In particular, the authors generated a library comprising linear, mono- and bi-cyclic peptides, demonstrating that the bicyclic variants exhibited superior performance in terms of both binding affinity and stability. Other recent examples of the use of the RaPID platform include the work of Magiera-Mularz et al. on the identification of immune checkpoint inhibitors targeting the PD-1/PD-L1 pathway [105], and Suga and collaborators on the discovery of both inhibitors and stabilizers of various PPIs [106,107,108].

#### 3.3.4. Split-and-Pool Techniques

**Figure 12:**
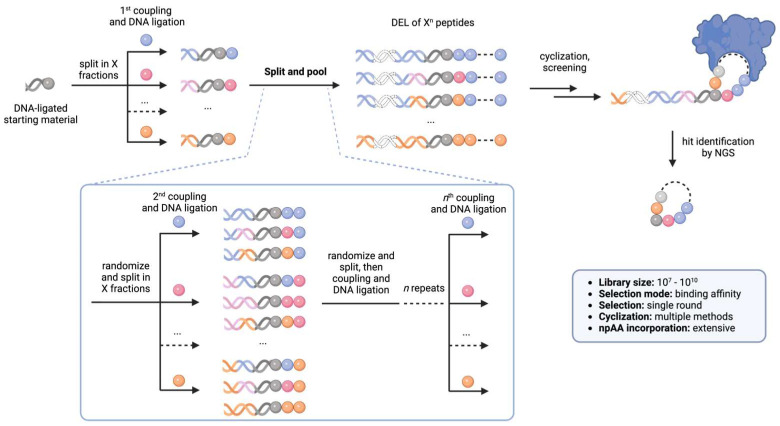
Schematization of the preparation of DNA-encoded libraries (DELs) with the split-and-pool technique. The starting material is ligated to a DNA fragment, split into X fractions, each of which is coupled with a different amino acid. Concurrently, a DNA barcode is ligated. The products are mixed together and then split again in X fractions, each of which is subjected to a second coupling reaction and addition of a DNA fragment. These steps are repeated *n* times to yield a DEL containing (theoretically) X^n^ different peptides, which can be cyclized by different methods and screened against the target. The hit(s) can be identified by sequencing the DNA barcodes.

Split-and-pool approaches are based on combinatorial chemistry and constitute an alternative to the display technologies discussed so far [46,88,109]. These techniques can either involve immobilization on a simple solid support, as in the one-bead-one-compound (OBOC) method [110,111], or derivatization with a nucleotide tag, as in DNA-encoded-libraries (DELs) [88,112]. Either way, the core idea is the same: the starting material (with or without the nucleotide tag) is split into X portions, each of which is reacted with an AA (either proteinogenic or not); then the products are pooled, mixed, split again and subjected to a new round of chemical synthesis. This process is repeated *n* times to generate a random library composed of X^n^ peptides, which is then screened against an immobilized target to select the hit(s) according to the binding affinity. In DELs, each addition of a building block is performed along with DNA-barcode ligation (Figure 12), so that the hit compound(s) can be identified by next-generation sequencing (NGS). On the other hand, OBOC is less expensive as it does not require DNA barcodes, but demands a more complex hit identification through MS/MS analysis. In any case, the split-and-pool techniques allow access to a vast chemical space, even with a few rounds, thanks to the fact that they rely on synthetic chemistry rather than display platforms, allowing easy incorporation of diverse npAAs.

The OBOC approach was employed by Liu et al. to identify a macrocyclic inhibitor of the interaction between calcineurin (Cn) and the nuclear factor of activated T cells (NFAT), endowed with a *K*_D_ < 1 μM and therapeutic potential as an immunosuppressive agent [113]. Similarly, Dewan et al. reported the discovery of an HIV-1 capsid (CA)–human lysyl-tRNA synthetase (hLysRS) interaction inhibitor with nanomolar *K*_D_ and in vitro IC_50_ of ~1 µM [114]. Another OBOC screening campaign led to the identification of potential anticancer agents targeting the K-Ras–effector interaction, as reported by Briesewitz and Pei [115,116]. More recently, a DEL screening was employed by Silvestri et al. to discover a nonapeptide inhibitor of the p53/MDM2 interaction (one of the most investigated PPIs and a driver in oncogenesis) with potency in the nanomolar range [117].

### 3.4. Computational and AI-Based Design of Cyclic Peptides

Computational methods leverage the data accumulated over decades of experimental structural biology, providing complementary strategies that reduce the experimental workload through synergistic integration with these datasets. While the discovery methods discussed above—based on iterative rounds of experimental screening—are limited by high cost, time consumption and restricted chemical space, in silico approaches enable iterative computational design and optimization of hits prior to experimental validation, thereby reducing the number of peptides that need to be synthesized and tested to identify a lead compound. Furthermore, computational methods allow (in principle) easier access to npAAs, thus facilitating the exploration of a wider and more diverse chemical space, although it must be noted that even the most recent AI models still suffer some limitations in handling npAAs [24,118].

Nowadays, in silico approaches can be roughly divided into physics-based methods and AI or data-driven methods [24,118]. The former includes molecular docking, molecular dynamics (MD), free energy calculations and quantum mechanics; they are based on explicit modeling of molecular structures and their interactions based on physical principles and are physically grounded and mechanistically interpretable, but they are also computationally demanding. The latter include AlphaFold-derived models, protein language models (PLMs), graph neural networks and generative models; they learn statistical patterns from large datasets, have a higher throughput and are increasingly employed in de novo ligand design and virtual screening, although their predictions are generally less mechanistically interpretable. Each of these tools can be employed for different specific purposes and at different stages in the discovery of cyclic peptide therapeutics, from de novo design to pharmacokinetic predictions. However, the optimal strategy in modern drug discovery is to use them synergistically within a “closed-loop” framework, in which generative models design molecular backbones that are subsequently validated through physics-based approaches (e.g., molecular dynamics simulations) to identify and eliminate potential “structural hallucinations”, i.e., de novo-generated structures that are geometrically complementary but thermodynamically unstable. The resulting data are then fed back into the generative models for iterative refinement and optimization. Further integration with experimental validation enhances the reliability of these computational approaches and helps reduce attrition rates.

#### 3.4.1. Structure Prediction

Structural prediction of peptides lacking experimental 3D data is crucial for drug development, although challenging due to cyclic constraints and conformational flexibility.

Traditional predictions often involve physics-based methods with parametrized force fields (e.g., molecular dynamics, conformational sampling, protein structure templates and secondary structure building) and provide detailed insights, although they are computationally expensive [33]. The development of the software Rosetta by Baker and co-workers in the 1990s is considered a milestone in the field of de novo protein design, thanks to its ability to reproduce the fundamental forces underlying protein folding [119,120]. More recently, the same research group modified their software to enable design of cyclic peptides and insertion of both D and L amino acids [121,122].

On the other hand, deep learning approaches rely on large datasets of known protein structures to provide sequence-based predictions with high accuracy and efficiency. The most notable example is AlphaFold [123], which demonstrated outstanding accuracy in predicting protein 3D structures, an achievement that was recognized with the 2024 Nobel Prize in Chemistry to Baker, Hassabis and Jumper [124]. Sequence-based PLMs represent a complementary platform, as they extract information directly from amino acid sequences without explicitly predicting 3D structures [125]. Despite these remarkable advancements, the development of dedicated models for cyclic peptides is limited by scarce training data for this class of compounds. Hybrid approaches (e.g., APPTEST [126], StrEAMM [127], and CRiSP [128]) were therefore conceived to address this limitation by integrating conventional structure prediction methods with AI models. More recently, fully AI-based models specifically developed for cyclic peptide structure prediction have begun to emerge. Deep learning models derived from AlphaFold such as AfCycDesign [129], HighFold [130] and AlphaFold3 [131] are examples of such tools, whereas PepSeA [132] and PepBAN [133] are representative of PLM-derived platforms.

Incorporating npAAs into these models is one of the main challenges today. While AfCycDesign [129] requires integration with physics-based methods to achieve this goal, second- and third-generation models (e.g., NCPepFold [134], CyclicBoltz1 [135], HighFold2 [136] and HighFold3 [137]) aim to solve the problem by using atomic-scale representations. One of the most recent achievements in this field is represented by the work of Wu and Zou, who accurately predicted the 3D structure of small cyclic peptides incorporating noncanonical structures, both in terms of cyclization linkages and amino acid side chains and backbones [138].

#### 3.4.2. Ligand Design

Another task that computational and AI tools can address is the design of novel ligands. Two main approaches can be adopted: template-based design and de novo design. The first takes advantage of available structural information (e.g., from known ligands, binding epitopes or ligand–receptor complex interfaces) to guide the optimization of cyclic peptides [118,139,140]. Molecular dynamic (MD) simulations and Rosetta are essential tools in this field, and they were used to discover selective ligands of histone deacetylases [141] and the New Delhi metallo-β-lactamase 1 [142].

In de novo design, novel cyclic peptide sequences are generated computationally to target proteins without known ligands or binding epitopes [143]. New sequences and scaffolds can be explored using computational and generative AI methods, including random evolutionary algorithms [144], hotspot identification and fragment growth (e.g., Des3PI [145] and CYC_BUILDER [146]), AlphaFold-based tools (such as AfDesign-cyclic [147] and EvoBind2 [148]), and the diffusion-based model RFpeptides [149]. Among these, the methods based on generative AI represent the most recent advance in the field, and their effectiveness has been proven in recent publications. For example, Wu et al. combined controlled generative modeling and discriminative modeling based on advanced deep learning to identify cyclic peptides targeting interleukin-17C (IL-17C), a cytokine involved in inflammatory diseases [150]. Xu et al. reported on the discovery of cyclic peptides targeting the intracellular protein KRAS (a GTPase involved in several cancers). The authors developed a model that combines hierarchical editing language for macromolecules (HELM) representation and a generative pre-trained transformer (GPT) with reinforcement learning. This model, named HELM-GPT, was able to co-optimize both binding affinity and cell permeability [151]. Another model, i.e., the aforementioned AfCycDesign, was able not only to predict with high accuracy the structures of cyclic peptides deposited in the Protein Data Bank (PDB), but also to redesign macrocyclic backbones capable of folding into desired structures, enabling de novo design of peptide binders for selected targets [129]. Finally, Li et al. employed EvoBind2 to design both linear and cyclic peptides targeting a semi-synthetic ribonuclease (PDB ID: 1SSC), used as a proof-of-concept model protein [148]. All of these approaches were validated through wet-lab experiments, thus supporting their viability.

Overall, in silico design techniques enable rational optimization of affinity and specificity, although they are still limited by sampling inefficiency, energy function inaccuracies, computational costs and restricted chemical space (especially for noncanonical amino acids, which still represent a major challenge). AI-based generative design is often based on a methodology called “hallucination”, in which peptide sequences are iteratively optimized toward a desired structural or functional objective (e.g., interaction with a target protein) based on AI-predicted structures. Starting from random sequences, mutations that improve the objective are retained until a novel candidate is generated. However, the resulting designs may not always be thermodynamically stable or experimentally viable, requiring further physics-based and experimental validation [147,152,153,154,155].

#### 3.4.3. Virtual Screening (VS)

Modern VS integrates docking, free energy calculations, MD and machine learning, enabling high-throughput evaluation of large cyclic peptide libraries to prioritize potentially active candidates for experimental validation. By identifying likely failures at an early stage, VS also helps reduce research and development costs and accelerates overall timelines.

VS methods are generally classified as ligand-based or structure-based: the former prioritize compounds based on their similarity to known active ligands, whereas the latter evaluate the complementarity between candidate molecules and the target binding surface [156,157,158]. The ligand-based approach was used by Duffy et al. to screen a combinatorial virtual library of cyclic peptides against a pharmacophore model derived from known ligands, eventually identifying a lead-like thrombin inhibitor [159]. Conversely, the structure-based approach was used by Chen et al. to identify cyclic peptides able to bind the ATAD2B protein, using a combination of molecular docking and MD, further refined by free energy calculations [160].

While these traditional VS approaches offer clear advantages—such as cost-effectiveness and efficiency, especially in comparison to experimental HTS—they also present some limitations: the exploration of large chemical spaces remains computationally demanding and often requires approximations that can affect accuracy. For example, they often consider rigid target conformations only, overlooking the dynamic structural changes that are crucial in PPIs and protein–cyclic peptide interactions, leading to a high rate of false positives [161]. Moreover, binding energy estimates derived from traditional physics-based methods (e.g., molecular docking, MM-GBSA, free energy perturbation) rely on explicit modeling of both 3D structure and molecular interactions between the cyclic peptide and its target, providing physically interpretable results, but at high computational cost. In addition, these methods rely heavily on detailed, target-specific knowledge and quality of starting structure, meaning that protocols optimized for one system are not easily transferable to others, thereby limiting their broader applicability in cyclic peptide drug discovery [118].

On the other hand, recent progress in machine learning has introduced a powerful addition to VS, mitigating some of these limitations. Exploiting large experimental datasets, AI-based models can learn complex relationships between molecular features and biological activity, enabling fast prediction of binding affinity and drug-like properties for cyclic peptide candidates. Unlike conventional methods, they do not strictly depend on high-resolution structural information or extensive target-specific expertise, and their ability to capture intricate patterns from sequence to structure and function can substantially improve screening performance [118]. Examples of AI tools used in VS campaigns include PepExplainer [162], PepScaf [163] and AlphaFold2 [164,165,166]. However, while AlphaFold-based methods provide rich structural information at a relatively high computational cost, PLMs (e.g., ESMFold [167,168], ProtT5 [169]) are much faster and more scalable, as they operate directly on the amino acid sequence without accurate 3D structure prediction. However, also in these cases, one must be aware of potential pitfalls: when dealing with PPIs and cyclic peptides there is a risk of using experimental datasets that are relatively small and biased toward natural amino acids. Therefore, the best approach to leverage computational advances in cyclic peptide drug discovery is to integrate the different available tools, for example, starting with PLMs to screen large peptide sequences, then applying AI-based structure prediction models (e.g., AlphaFold-derived methods) for more accurate structural modeling of the top candidates, and finally refining the results with physics-based methods, thus increasing computational demand at each step on a progressively restricted set of candidates. In addition, integration between virtual screening and experimental validation with iterative, real-time feedback is crucial for model validation and subsequent optimization.

#### 3.4.4. Prediction of Pharmacokinetics and Drug-likeness

Computational approaches are increasingly being used for predicting key pharmacokinetic (PK) parameters and drug-likeness in early phases of drug discovery. Absorption, distribution, metabolism, excretion (ADME) and toxicity issues constitute one of the major risks of attrition for any drug candidate, including cyclic peptides [170,171,172,173]. Therefore, assessing those properties in advance is essential for identifying potential liabilities and streamlining the optimization process, in accordance with the paradigm “fail fast, fail cheap”. While experimental evaluation of the pharmacokinetic profile is costly, time-consuming and has a limited throughput, in silico methods offer a cheaper and quicker complement that can enhance the success rate of the drug discovery process. For cyclic peptides, the most crucial PK properties are membrane permeability (which influences oral bioavailability and access to intracellular targets) and half-life (as peptide-based drugs are susceptible to rapid metabolism). These can be predicted with data-driven and statistical models, physics-based methods and machine learning models.

In particular, the prediction of membrane permeability for cyclic peptides follows principles that differ from those governing small molecules, whose permeability is typically assessed based on parameters such as molecular size, hydrogen-bonding capacity, and logP (o/w). As discussed previously, the behavior of cyclic peptides is better explained by the “chameleon hypothesis,” which reflects their ability to adopt different conformations in response to the surrounding environment [32,174]. Statistical approaches like quantitative structure–property relationship (QSPR) enable rapid predictions but depend strongly on experimental datasets and often fail to capture 3D structure and dynamics [175,176]. Physics-based models and MD simulations provide mechanistic insights into membrane permeation, including conformational changes and energy barriers, although they require calibration and are computationally demanding [177,178,179]. Machine learning methods (e.g., random forest regression models) improve the prediction of cyclic peptide membrane permeability by combining higher speed with greater accuracy, although their performance remains limited by data scarcity and structural complexity. The development of dedicated datasets such as CycPeptMPDB [180] has helped address these issues, enabling the training of more robust models. Leveraging these curated datasets, recent multimodal approaches (e.g., Multi_CycGT [181], CycPeptMP [182], MuCoCP [183] and PEGASUS [184]) integrate structural, physicochemical, and sequence information, leading to enhanced predictive performance. However, limitations persist due to incomplete 3D structural representation and the prevalence of npAAs in available datasets [185]. Emerging structure prediction tools like AlphaFold3 [131] and RoseTTAFold All-Atom [186], as well as the chemical language model PeptideCLM [187], are expected to further improve model accuracy in this field.

Prediction of peptide half-life, on the other hand, relies on both rule-based and machine learning approaches. N-end rule models such as ProtLifePred and ProtParam estimate stability from N-terminal residues (e.g., Arg and Lys activate ubiquitin–proteasome degradation), but are limited by their simplified view and lack of structural and physiological context [188,189]. Machine learning methods improve predictive performance, particularly for blood stability, yet remain constrained by (i) limited and heterogeneous datasets and (ii) their inability to predict half-life variability across species and organs, or upon peptide modifications [190,191,192]. Recent advances address these issues through the development of high-quality, diverse datasets and more sophisticated models that integrate enzymatic cleavage features and multimodal descriptors, including 3D structure, PLMs and graph neural networks. Furthermore, recent advancements in the development of multimodal frameworks are enabling more accurate assessment of safety and toxicity [193,194]. These improvements have significantly enhanced prediction accuracy across different biological contexts, effectively supporting peptide drug development [192,195,196].

## 4. Conclusions

Cyclic peptides have established themselves as a versatile and increasingly viable modality for targeting PPIs, effectively bridging the gap between small molecules and biologics. Their ability to mimic protein epitopes, combined with enhanced conformational stability and improved pharmacological properties, makes them particularly well suited to address the challenges posed by PPI interfaces [25,31,34]. The increasing number of cyclic peptides that reached regulatory approval, as well as the progression of several others into clinical trials—including orally bioavailable candidates—further highlights the therapeutic value of this class of molecules [6,18,98,118].

The diversity of available discovery strategies, from natural products to rational design and high-throughput screening, provides multiple approaches for the identification of bioactive scaffolds. Importantly, these strategies are not mutually exclusive but are most effective when integrated within iterative workflows that combine experimental and computational methods. Furthermore, the fundamental importance of multi-parameter optimization and rigorous translational validation is being increasingly recognized: besides binding affinity, early evaluation of in-cell target engagement, pathway modulation, cell permeability, solubility and metabolic stability is crucial to reduce attrition rates. Most cyclic peptides fall in the “beyond-rule-of-5” chemical space, thus posing significant challenges in their pharmacokinetic optimization [175,197]. In this context, recent advances in AI-driven tools are gaining increasing importance, enabling more efficient exploration of chemical space, improved structure prediction, and more accurate estimation of drug-like properties. Despite these advances, several challenges remain, including limited availability of high-quality datasets (unbiased toward natural or non-natural amino acids), difficulties in accurately predicting permeability and in vivo stability, and the intrinsic complexity of PPI interfaces. Addressing these limitations is expected to shape future research directions. These include expanding the scope and throughput of wet-lab screening methods (with platforms that can accurately mimic cellular environment), effective incorporation of npAAs (both in synthetic and computational methodologies) and curation of better experimental datasets, suitable for the development of AI models. These represent just a few examples of the efforts that will likely drive future advances in the field, requiring continued development of hybrid methodologies, improved data integration, and closer combination of in silico predictions with experimental validation [25,88,89].

Finally, it is worth noting that most of current research focuses on PPI inhibition, whereas the stabilization of beneficial PPIs is equally promising but remains relatively underexplored. Cyclic peptides may serve this purpose as well, representing a valuable starting point for the development of molecular glues, proximity inducers and stabilizers of ternary complexes [198].

Overall, the field is rapidly evolving, and cyclic peptides are expected to play a pivotal role in expanding the druggable proteome. Continued innovation in design strategies, screening technologies, and computational methods will be essential to fully unlock their therapeutic potential.

## Data Availability

No new data were created or analyzed in this study. Data sharing is not applicable to this article.

## References

[1] Greenblatt J.F., Alberts B.M., Krogan N.J. (2024). Discovery and Significance of Protein-Protein Interactions in Health and Disease. Cell.

[2] Wang S., Wu R., Lu J., Jiang Y., Huang T., Cai Y. (2022). Protein-protein Interaction Networks as Miners of Biological Discovery. Proteomics.

[3] Ramos R.H., de Oliveira Lage Ferreira C., Simao A. (2024). Human Protein–Protein Interaction Networks: A Topological Comparison Review. Heliyon.

[4] Marković V., Szczepańska A., Berlicki Ł. (2024). Antiviral Protein–Protein Interaction Inhibitors. J. Med. Chem..

[5] Shin W.-H., Kumazawa K., Imai K., Hirokawa T., Kihara D. (2020). Current Challenges and Opportunities in Designing Protein–Protein Interaction Targeted Drugs. AABC.

[6] Philippe G.J.B., Craik D.J., Henriques S.T. (2021). Converting Peptides into Drugs Targeting Intracellular Protein–Protein Interactions. Drug Discov. Today.

[7] Jiang Y., Chen M., Nie H., Yuan Y. (2019). PD-1 and PD-L1 in Cancer Immunotherapy: Clinical Implications and Future Considerations. Hum. Vaccines Immunother..

[8] Center for Drug Evaluation and Research-FDA (2023). Approves Dostarlimab-Gxly with Chemotherapy for Endometrial Cancer.

[9] Center for Drug Evaluation and Research-FDA (2025). Approves Retifanlimab-Dlwr with Carboplatin and Paclitaxel and as a Single Agent for Squamous Cell Carcinoma of the Anal Canal.

[10] Center for Drug Evaluation and Research-FDA (2024). Approves Toripalimab-Tpzi for Nasopharyngeal Carcinoma.

[11] Center for Drug Evaluation and Research-FDA (2026). Drug Trials Snapshots: TEVIMBRA.

[12] Tevimbra|European Medicines Agency (EMA). https://www.ema.europa.eu/en/medicines/human/EPAR/tevimbra.

[13] Center for Drug Evaluation and Research-FDA (2024). Approves Cosibelimab-Ipdl for Metastatic or Locally Advanced Cutaneous Squamous Cell Carcinoma.

[14] Muccini C., Canetti D., Castagna A., Spagnuolo V. (2022). Efficacy and Safety Profile of Fostemsavir for the Treatment of People with Human Immunodeficiency Virus-1 (HIV-1): Current Evidence and Place in Therapy. Drug Des. Devel Ther..

[15] Heidary M., Shariati S., Nourigheimasi S., Khorami M., Moradi M., Motahar M., Bahrami P., Akrami S., Kaviar V.H. (2024). Mechanism of Action, Resistance, Interaction, Pharmacokinetics, Pharmacodynamics, and Safety of Fostemsavir. BMC Infect. Dis..

[16] Center for Drug Evaluation and Research-FDA (2019). Venetoclax (Venclexta) Tablets.

[17] Cao Q., Wu X., Zhang Q., Gong J., Chen Y., You Y., Shen J., Qiang Y., Cao G. (2023). Mechanisms of Action of the BCL-2 Inhibitor Venetoclax in Multiple Myeloma: A Literature Review. Front. Pharmacol..

[18] Guerlavais V., Sawyer T.K., Carvajal L., Chang Y.S., Graves B., Ren J.-G., Sutton D., Olson K.A., Packman K., Darlak K. (2023). Discovery of Sulanemadlin (ALRN-6924), the First Cell-Permeating, Stabilized α-Helical Peptide in Clinical Development. J. Med. Chem..

[19] Milroy L.-G., Grossmann T.N., Hennig S., Brunsveld L., Ottmann C. (2014). Modulators of Protein–Protein Interactions. Chem. Rev..

[20] Monti A., Vitagliano L., Caporale A., Ruvo M., Doti N. (2023). Targeting Protein–Protein Interfaces with Peptides: The Contribution of Chemical Combinatorial Peptide Library Approaches. Int. J. Mol. Sci..

[21] Wang H., Dawber R.S., Zhang P., Walko M., Wilson A.J., Wang X. (2021). Peptide-Based Inhibitors of Protein–Protein Interactions: Biophysical, Structural and Cellular Consequences of Introducing a Constraint. Chem. Sci..

[22] Tsomaia N. (2015). Peptide Therapeutics: Targeting the Undruggable Space. Eur. J. Med. Chem..

[23] Trisciuzzi D., Villoutreix B.O., Siragusa L., Baroni M., Cruciani G., Nicolotti O. (2023). Targeting Protein-Protein Interactions with Low Molecular Weight and Short Peptide Modulators: Insights on Disease Pathways and Starting Points for Drug Discovery. Expert Opin. Drug Discov..

[24] Hashemi Z.S., Zarei M., Fath M.K., Ganji M., Farahani M.S., Afsharnouri F., Pourzardosht N., Khalesi B., Jahangiri A., Rahbar M.R. (2021). In Silico Approaches for the Design and Optimization of Interfering Peptides Against Protein–Protein Interactions. Front. Mol. Biosci..

[25] Cheng J., Zhou J., Kong L., Wang H., Zhang Y., Wang X., Liu G., Chu Q. (2023). Stabilized Cyclic Peptides as Modulators of Protein–Protein Interactions: Promising Strategies and Biological Evaluation. RSC Med. Chem..

[26] Paulussen F.M., Grossmann T.N. (2023). Peptide-Based Covalent Inhibitors of Protein–Protein Interactions. J. Pept. Sci..

[27] Lee A.C.-L., Harris J.L., Khanna K.K., Hong J.-H. (2019). A Comprehensive Review on Current Advances in Peptide Drug Development and Design. Int. J. Mol. Sci..

[28] Wang J., Zheng P., Yu J., Yang X., Zhang J. (2024). Rational Design of Small-Sized Peptidomimetic Inhibitors Disrupting Protein–Protein Interaction. RSC Med. Chem..

[29] Zhang H., Chen S. (2022). Cyclic Peptide Drugs Approved in the Last Two Decades (2001–2021). RSC Chem. Biol..

[30] Buckton L.K., Rahimi M.N., McAlpine S.R. (2021). Cyclic Peptides as Drugs for Intracellular Targets: The Next Frontier in Peptide Therapeutic Development. Chemistry.

[31] González-Muñiz R., Bonache M.Á., Pérez De Vega M.J. (2021). Modulating Protein–Protein Interactions by Cyclic and Macrocyclic Peptides. Prominent Strategies and Examples. Molecules.

[32] Whitty A., Zhong M., Viarengo L., Beglov D., Hall D.R., Vajda S. (2016). Quantifying the Chameleonic Properties of Macrocycles and Other High-Molecular-Weight Drugs. Drug Discov. Today.

[33] Damjanovic J., Miao J., Huang H., Lin Y.-S. (2021). Elucidating Solution Structures of Cyclic Peptides Using Molecular Dynamics Simulations. Chem. Rev..

[34] Buyanova M., Pei D. (2022). Targeting Intracellular Protein–Protein Interactions with Macrocyclic Peptides. Trends Pharmacol. Sci..

[35] Choi J.-S., Joo S.H. (2020). Recent Trends in Cyclic Peptides as Therapeutic Agents and Biochemical Tools. Biomol. Ther..

[36] Ji X., Nielsen A.L., Heinis C. (2024). Cyclic Peptides for Drug Development. Angew. Chem. Int. Ed..

[37] Wu C., Wang H. (2023). Recent Progress on Cyclic Peptides’ Assembly and Biomedical Applications. ChemBioChem.

[38] Martian P.C., Tertis M., Leonte D., Hadade N., Cristea C., Crisan O. (2025). Cyclic Peptides: A Powerful Instrument for Advancing Biomedical Nanotechnologies and Drug Development. J. Pharm. Biomed. Anal..

[39] Ramadhani D., Maharani R., Gazzali A.M., Muchtaridi M. (2022). Cyclic Peptides for the Treatment of Cancers: A Review. Molecules.

[40] van Neer R.H.P., Dranchak P.K., Aitha M., Liu L., Carlson E.K., Jacobsen I.E., Battaile K., Fang Y., Tao D., Rai G. (2025). Active- and Allosteric-Site Cyclic Peptide Inhibitors of Secreted M. Tuberculosis Chorismate Mutase. ACS Infect. Dis..

[41] Chang A.W., Dowd S.E., Brackee G., Fralick J.A., Vediyappan G. (2022). Inhibition of Staphylococcus Aureus Biofilm Formation by Gurmarin, a Plant-Derived Cyclic Peptide. Front. Cell. Infect. Microbiol..

[42] D’Aloisio V., Dognini P., Hutcheon G.A., Coxon C.R. (2021). PepTherDia: Database and Structural Composition Analysis of Approved Peptide Therapeutics and Diagnostics. Drug Discov. Today.

[43] Knox C., Wilson M., Klinger C.M., Franklin M., Oler E., Wilson A., Pon A., Cox J., Chin N.E.L., Strawbridge S.A. (2024). DrugBank 6.0: The DrugBank Knowledgebase for 2024. Nucleic Acids Res..

[44] Fang P., Pang W.-K., Xuan S., Chan W.-L., Leung K.C.-F. (2024). Recent Advances in Peptide Macrocyclization Strategies. Chem. Soc. Rev..

[45] Bechtler C., Lamers C. (2021). Macrocyclization Strategies for Cyclic Peptides and Peptidomimetics. RSC Med. Chem..

[46] Shinbara K., Liu W., van Neer R.H.P., Katoh T., Suga H. (2020). Methodologies for Backbone Macrocyclic Peptide Synthesis Compatible with Screening Technologies. Front. Chem..

[47] Wang W., Khojasteh S.C., Su D. (2021). Biosynthetic Strategies for Macrocyclic Peptides. Molecules.

[48] Sohrabi C., Foster A., Tavassoli A. (2020). Methods for Generating and Screening Libraries of Genetically Encoded Cyclic Peptides in Drug Discovery. Nat. Rev. Chem..

[49] Jacob B., Vogelaar A., Cadenas E., Camarero J.A. (2022). Using the Cyclotide Scaffold for Targeting Biomolecular Interactions in Drug Development. Molecules.

[50] Troeira Henriques S., Lawrence N., Kan M.-W., Malins L.R., Craik D.J. (2025). Cell-Penetrating Cyclic and Disulfide-Rich Peptides Are Privileged Molecular Scaffolds for Intracellular Targeting. Biochemistry.

[51] Liu L., Yang L., Cao S., Gao Z., Yang B., Zhang G., Zhu R., Wu D. (2024). CyclicPepedia: A Knowledge Base of Natural and Synthetic Cyclic Peptides. Brief. Bioinform..

[52] de Veer S.J., Kan M.-W., Craik D.J. (2019). Cyclotides: From Structure to Function. Chem. Rev..

[53] Hyun Y. (2025). Cyclotides as Novel Plant-Derived Scaffolds for Orally Active Cyclic Peptide Therapeutics. Mol. Cells.

[54] Philippe G.J.-B., Huang Y.-H., Mittermeier A., Brown C.J., Kaas Q., Ramlan S.R., Wang C.K., Lane D., Loewer A., Troeira Henriques S. (2024). Delivery to, and Reactivation of, the P53 Pathway in Cancer Cells Using a Grafted Cyclotide Conjugated with a Cell-Penetrating Peptide. J. Med. Chem..

[55] Koehbach J., Muratspahić E., Ahmed Z.M., White A.M., Tomašević N., Durek T., Clark R.J., Gruber C.W., Craik D.J. (2024). Chemical Synthesis of Grafted Cyclotides Using a “Plug and Play” Approach. RSC Chem. Biol..

[56] Najmi A., Javed S.A., Al Bratty M., Alhazmi H.A. (2022). Modern Approaches in the Discovery and Development of Plant-Based Natural Products and Their Analogues as Potential Therapeutic Agents. Molecules.

[57] Clark R.C., Lee S.Y., Searcey M., Boger D.L. (2009). The Isolation, Total Synthesis and Structure Elucidation of Chlorofusin, a Natural Product Inhibitor of the P53–MDM2 Protein–Protein Interaction. Nat. Prod. Rep..

[58] Kersten R.D., Weng J.-K. (2018). Gene-Guided Discovery and Engineering of Branched Cyclic Peptides in Plants. Proc. Natl. Acad. Sci. USA.

[59] Muthuraj R., Chandrasekaran J. (2026). Nature Meets Machine: The AI Renaissance in Natural Product Drug Discovery. Nat. Prod. Bioprospect..

[60] Ory L., Nazih E.-H., Daoud S., Mocquard J., Bourjot M., Margueritte L., Delsuc M.-A., Bard J.-M., Pouchus Y.F., Bertrand S. (2019). Targeting Bioactive Compounds in Natural Extracts—Development of a Comprehensive Workflow Combining Chemical and Biological Data. Anal. Chim. Acta.

[61] Zhang J., Yuan J., Li Z., Fu C., Xu M., Yang J., Jiang X., Zhou B., Ye X., Xu C. (2021). Exploring and Exploiting Plant Cyclic Peptides for Drug Discovery and Development. Med. Res. Rev..

[62] Hostetler M.A., Smith C., Nelson S., Budimir Z., Modi R., Woolsey I., Frerk A., Baker B., Gantt J., Parkinson E.I. (2021). Synthetic Natural Product Inspired Cyclic Peptides. ACS Chem. Biol..

[63] Yang X., Lennard K.R., He C., Walker M.C., Ball A.T., Doigneaux C., Tavassoli A., van der Donk W.A. (2018). A Lanthipeptide Library Used to Identify a Protein–Protein Interaction Inhibitor. Nat. Chem. Biol..

[64] Le T., Jeanne Dit Fouque K., Santos-Fernandez M., Navo C.D., Jiménez-Osés G., Sarksian R., Fernandez-Lima F.A., van der Donk W.A. (2021). Substrate Sequence Controls Regioselectivity of Lanthionine Formation by ProcM. J. Am. Chem. Soc..

[65] Wang H., Liu C., Deng L. (2018). Enhanced Prediction of Hot Spots at Protein-Protein Interfaces Using Extreme Gradient Boosting. Sci. Rep..

[66] Monteleone S., Fedorov D.G., Townsend-Nicholson A., Southey M., Bodkin M., Heifetz A. (2022). Hotspot Identification and Drug Design of Protein–Protein Interaction Modulators Using the Fragment Molecular Orbital Method. J. Chem. Inf. Model..

[67] Pelay-Gimeno M., Glas A., Koch O., Grossmann T.N. (2015). Structure-Based Design of Inhibitors of Protein–Protein Interactions: Mimicking Peptide Binding Epitopes. Angew. Chem. Int. Ed..

[68] Bullock B.N., Jochim A.L., Arora P.S. (2011). Assessing Helical Protein Interfaces for Inhibitor Design. J. Am. Chem. Soc..

[69] Fairlie D.P., Dantas de Araujo A. (2016). Stapling Peptides Using Cysteine Crosslinking. Pept. Sci..

[70] Li X., Chen S., Zhang W.-D., Hu H.-G. (2020). Stapled Helical Peptides Bearing Different Anchoring Residues. Chem. Rev..

[71] Schafmeister C.E., Po J., Verdine G.L. (2000). An All-Hydrocarbon Cross-Linking System for Enhancing the Helicity and Metabolic Stability of Peptides. J. Am. Chem. Soc..

[72] Walensky L.D., Bird G.H. (2014). Hydrocarbon-Stapled Peptides: Principles, Practice, and Progress. J. Med. Chem..

[73] Jedhe G.S., Arora P.S. (2021). Hydrogen Bond Surrogate Helices as Minimal Mimics of Protein α-Helices. Methods in Enzymology.

[74] Wang D., Liao W., Arora P.S. (2005). Enhanced Metabolic Stability and Protein-Binding Properties of Artificial α Helices Derived from a Hydrogen-Bond Surrogate: Application to Bcl-xL. Angew. Chem. Int. Ed..

[75] Bao J., Dong X.Y., Zhang J.Z.H., Arora P.S. (2009). Dynamical Binding of Hydrogen-Bond Surrogate Derived Bak Helices to Antiapoptotic Protein Bcl-xL. J. Phys. Chem. B.

[76] Douse C.H., Maas S.J., Thomas J.C., Garnett J.A., Sun Y., Cota E., Tate E.W. (2014). Crystal Structures of Stapled and Hydrogen Bond Surrogate Peptides Targeting a Fully Buried Protein–Helix Interaction. ACS Chem. Biol..

[77] Henchey L.K., Porter J.R., Ghosh I., Arora P.S. (2010). High Specificity in Protein Recognition by Hydrogen-Bond-Surrogate α-Helices: Selective Inhibition of the P53/MDM2 Complex. ChemBioChem.

[78] Ramírez-Alvarado M., Kortemme T., Blanco F.J., Serrano L. (1999). β-Hairpin and β-Sheet Formation in Designed Linear Peptides. Bioorganic Med. Chem..

[79] Robinson J.A. (2008). β-Hairpin Peptidomimetics: Design, Structures and Biological Activities. Acc. Chem. Res..

[80] Robinson J.A., DeMarco S., Gombert F., Moehle K., Obrecht D. (2008). The Design, Structures and Therapeutic Potential of Protein Epitope Mimetics. Drug Discov. Today.

[81] Lingard H., Han J.T., Thompson A.L., Leung I.K.H., Scott R.T.W., Thompson S., Hamilton A.D. (2014). Diphenylacetylene-Linked Peptide Strands Induce Bidirectional β-Sheet Formation. Angew. Chem. Int. Ed..

[82] Sawyer N., Arora P.S. (2018). Hydrogen Bond Surrogate Stabilization of β-Hairpins. ACS Chem. Biol..

[83] Nazzaro A., Lu B., Sawyer N., Watkins A.M., Arora P.S. (2023). Macrocyclic β-Sheets Stabilized by Hydrogen Bond Surrogates. Angew. Chem. Int. Ed..

[84] Hill T.A., Shepherd N.E., Diness F., Fairlie D.P. (2014). Constraining Cyclic Peptides To Mimic Protein Structure Motifs. Angew. Chem. Int. Ed..

[85] Adams Z.C., Silvestri A.P., Chiorean S., Flood D.T., Balo B.P., Shi Y., Holcomb M., Walsh S.I., Maillie C.A., Pierens G.K. (2023). Stretching Peptides to Generate Small Molecule β-Strand Mimics. ACS Cent. Sci..

[86] Wendt M., Bellavita R., Gerber A., Efrém N.-L., van Ramshorst T., Pearce N.M., Davey P.R.J., Everard I., Vazquez-Chantada M., Chiarparin E. (2021). Bicyclic β-Sheet Mimetics That Target the Transcriptional Coactivator β-Catenin and Inhibit Wnt Signaling. Angew. Chem. Int. Ed..

[87] Laxio Arenas J., Kaffy J., Ongeri S. (2019). Peptides and Peptidomimetics as Inhibitors of Protein–Protein Interactions Involving β-Sheet Secondary Structures. Curr. Opin. Chem. Biol..

[88] Colas K., Bindl D., Suga H. (2024). Selection of Nucleotide-Encoded Mass Libraries of Macrocyclic Peptides for Inaccessible Drug Targets. Chem. Rev..

[89] Li X., Craven T.W., Levine P.M. (2022). Cyclic Peptide Screening Methods for Preclinical Drug Discovery: Miniperspective. J. Med. Chem..

[90] Bakhshinejad B., Ghiasvand S. (2025). A Beautiful Bind: Phage Display and the Search for Cell-Selective Peptides. Viruses.

[91] Chen F., Pinnette N., Gao J. (2024). Strategies for the Construction of Multicyclic Phage Display Libraries. ChemBioChem.

[92] Oller-Salvia B., Chin J.W. (2019). Efficient Phage Display with Multiple Distinct Non-Canonical Amino Acids Using Orthogonal Ribosome-Mediated Genetic Code Expansion. Angew. Chem. Int. Ed. Engl..

[93] Carle V., Kong X.-D., Comberlato A., Edwards C., Díaz-Perlas C., Heinis C. (2021). Generation of a 100-Billion Cyclic Peptide Phage Display Library Having a High Skeletal Diversity. Protein Eng. Des. Sel..

[94] Wood D.W., Belfort M., Lennon C.W. (2023). Inteins—Mechanism of Protein Splicing, Emerging Regulatory Roles, and Applications in Protein Engineering. Front. Microbiol..

[95] Tavassoli A. (2017). SICLOPPS Cyclic Peptide Libraries in Drug Discovery. Curr. Opin. Chem. Biol..

[96] McDermott A., Windeln L.M., Valentine J.S.D., Baldassarre L., Foster A.D., Tavassoli A. (2024). Next Generation SICLOPPS Screening for the Identification of Inhibitors of the HIF-1α/HIF-1β Protein–Protein Interaction. ACS Chem. Biol..

[97] Lennard K.R., Gardner R.M., Doigneaux C., Castillo F., Tavassoli A. (2019). Development of a Cyclic Peptide Inhibitor of the P6/UEV Protein–Protein Interaction. ACS Chem. Biol..

[98] Josien H., Nair A.G., Ding F.-X., Guo Y., Chen Y.-H., Rao A.U., Liu J., Tong L., Sun Z., Lo M.M.-C. (2026). Discovery Process of Enlicitide, a Highly Engineered Macrocyclic Peptide Therapeutic, through Issue-Driven Fragment-Based Synthetic Assembly and SAR. J. Med. Chem..

[99] Merck Sharp & Dohme LLC (2026). Phase 3 Randomized, Placebo-Controlled Clinical Study to Evaluate the Efficacy and Safety of MK-0616 in Reducing Major Adverse Cardiovascular Events in Participants at High Cardiovascular Risk. https://clinicaltrials.gov/study/NCT06008756.

[100] Chiyoda A., Matsuo A., Yamano T., Tanada M. (2026). Structure–Activity Relationship Analysis of Macrocyclic Peptide RAS Inhibitors: Spotlight on the Solvent-Exposed Region. ACS Med. Chem. Lett..

[101] Chugai Pharmaceutical (2025). A Phase 1 Open-Label, Dose-Escalation and Cohort Expansion Study of LUNA18 Monotherapy and Combination Therapy in Patients with Locally Advanced or Metastatic Solid Tumors. https://clinicaltrials.gov/study/NCT05012618.

[102] Goto Y., Suga H. (2021). The RaPID Platform for the Discovery of Pseudo-Natural Macrocyclic Peptides. Acc. Chem. Res..

[103] Takatsuji R., Shinbara K., Katoh T., Goto Y., Passioura T., Yajima R., Komatsu Y., Suga H. (2019). Ribosomal Synthesis of Backbone-Cyclic Peptides Compatible with In Vitro Display. J. Am. Chem. Soc..

[104] Villequey C., Zurmühl S.S., Cramer C.N., Bhusan B., Andersen B., Ren Q., Liu H., Qu X., Yang Y., Pan J. (2024). An Efficient mRNA Display Protocol Yields Potent Bicyclic Peptide Inhibitors for FGFR3c: Outperforming Linear and Monocyclic Formats in Affinity and Stability. Chem. Sci..

[105] Magiera-Mularz K., Skalniak L., Zak K.M., Musielak B., Rudzinska-Szostak E., Berlicki Ł., Kocik J., Grudnik P., Sala D., Zarganes-Tzitzikas T. (2017). Bioactive Macrocyclic Inhibitors of the PD-1/PD-L1 Immune Checkpoint. Angew. Chem. Int. Ed..

[106] Nitsche C., Passioura T., Varava P., Mahawaththa M.C., Leuthold M.M., Klein C.D., Suga H., Otting G. (2019). De Novo Discovery of Nonstandard Macrocyclic Peptides as Noncompetitive Inhibitors of the Zika Virus NS2B-NS3 Protease. ACS Med. Chem. Lett..

[107] Passioura T., Watashi K., Fukano K., Shimura S., Saso W., Morishita R., Ogasawara Y., Tanaka Y., Mizokami M., Sureau C. (2018). De Novo Macrocyclic Peptide Inhibitors of Hepatitis B Virus Cellular Entry. Cell Chem. Biol..

[108] Ito K., Sakai K., Suzuki Y., Ozawa N., Hatta T., Natsume T., Matsumoto K., Suga H. (2015). Artificial Human Met Agonists Based on Macrocycle Scaffolds. Nat. Commun..

[109] Lam K.S., Salmon S.E., Hersh E.M., Hruby V.J., Kazmierski W.M., Knapp R.J. (1991). A New Type of Synthetic Peptide Library for Identifying Ligand-Binding Activity. Nature.

[110] Krchnák V. (1997). The “One-Bead-One-Compound” Combinatorial Library Method. Chem. Rev..

[111] Lam K.S., Lehman A.L., Song A., Doan N., Enstrom A.M., Maxwell J., Liu R. (2003). Synthesis and Screening of “One-Bead One-Compound” Combinatorial Peptide Libraries. Methods in Enzymology.

[112] Plais L., Scheuermann J. (2022). Macrocyclic DNA-Encoded Chemical Libraries: A Historical Perspective. RSC Chem. Biol..

[113] Liu T., Qian Z., Xiao Q., Pei D. (2011). High-Throughput Screening of One-Bead-One-Compound Libraries: Identification of Cyclic Peptidyl Inhibitors against Calcineurin/NFAT Interaction. ACS Comb. Sci..

[114] Dewan V., Liu T., Chen K.-M., Qian Z., Xiao Y., Kleiman L., Mahasenan K.V., Li C., Matsuo H., Pei D. (2012). Cyclic Peptide Inhibitors of HIV-1 Capsid-Human Lysyl-tRNA Synthetase Interaction. ACS Chem. Biol..

[115] Wu X., Upadhyaya P., Villalona-Calero M.A., Briesewitz R., Pei D. (2013). Inhibition of Ras–Effector Interactions by Cyclic Peptides. Med. Chem. Commun..

[116] Upadhyaya P., Qian Z., Selner N.G., Clippinger S.R., Wu Z., Briesewitz R., Pei D. (2015). Inhibition of Ras Signaling by Blocking Ras–Effector Interactions with Cyclic Peptides. Angew. Chem. Int. Ed..

[117] Silvestri A.P., Zhang Q., Ping Y., Muir E.W., Zhao J., Chakka S.K., Wang G., Bray W.M., Chen W., Fribourgh J.L. (2023). DNA-Encoded Macrocyclic Peptide Libraries Enable the Discovery of a Neutral MDM2–P53 Inhibitor. ACS Med. Chem. Lett..

[118] Lin K., Zhang C., Bai R., Duan H. (2025). Cyclic Peptide Therapeutic Agents Discovery: Computational and Artificial Intelligence-Driven Strategies. J. Med. Chem..

[119] Han K.F., Baker D. (1996). Global Properties of the Mapping between Local Amino Acid Sequence and Local Structure in Proteins. Proc. Natl. Acad. Sci. USA.

[120] Simons K.T., Kooperberg C., Huang E., Baker D. (1997). Assembly of Protein Tertiary Structures from Fragments with Similar Local Sequences Using Simulated Annealing and Bayesian Scoring Functions. J. Mol. Biol..

[121] Bhardwaj G., Mulligan V.K., Bahl C.D., Gilmore J.M., Harvey P.J., Cheneval O., Buchko G.W., Pulavarti S.V.S.R.K., Kaas Q., Eletsky A. (2016). Accurate de Novo Design of Hyperstable Constrained Peptides. Nature.

[122] Hosseinzadeh P., Bhardwaj G., Mulligan V.K., Shortridge M.D., Craven T.W., Pardo-Avila F., Rettie S.A., Kim D.E., Silva D.-A., Ibrahim Y.M. (2017). Comprehensive Computational Design of Ordered Peptide Macrocycles. Science.

[123] Jumper J., Evans R., Pritzel A., Green T., Figurnov M., Ronneberger O., Tunyasuvunakool K., Bates R., Žídek A., Potapenko A. (2021). Highly Accurate Protein Structure Prediction with AlphaFold. Nature.

[124] Nobel Prize in Chemistry 2024. https://www.nature.com/collections/edjcfdihdi.

[125] Ekambaram S., Dokholyan N.V. (2026). Peptide-Based Drug Design Using Generative AI. Chem. Commun..

[126] Timmons P.B., Hewage C.M. (2021). APPTEST Is a Novel Protocol for the Automatic Prediction of Peptide Tertiary Structures. Brief. Bioinform..

[127] Hui T., Descoteaux M.L., Miao J., Lin Y.-S. (2023). Training Neural Network Models Using Molecular Dynamics Simulation Results to Efficiently Predict Cyclic Hexapeptide Structural Ensembles. J. Chem. Theory Comput..

[128] Liu Z.-L., Hu J.-H., Jiang F., Wu Y.-D. (2020). CRiSP: Accurate Structure Prediction of Disulfide-Rich Peptides with Cystine-Specific Sequence Alignment and Machine Learning. Bioinformatics.

[129] Rettie S.A., Campbell K.V., Bera A.K., Kang A., Kozlov S., Bueso Y.F., De La Cruz J., Ahlrichs M., Cheng S., Gerben S.R. (2025). Cyclic Peptide Structure Prediction and Design Using AlphaFold2. Nat. Commun..

[130] Zhang C., Zhang C., Shang T., Zhu N., Wu X., Duan H. (2024). HighFold: Accurately Predicting Structures of Cyclic Peptides and Complexes with Head-to-Tail and Disulfide Bridge Constraints. Brief. Bioinform..

[131] Abramson J., Adler J., Dunger J., Evans R., Green T., Pritzel A., Ronneberger O., Willmore L., Ballard A.J., Bambrick J. (2024). Accurate Structure Prediction of Biomolecular Interactions with AlphaFold 3. Nature.

[132] Baylon J.L., Ursu O., Muzdalo A., Wassermann A.M., Adams G.L., Spale M., Mejzlik P., Gromek A., Pisarenko V., Hancharyk D. (2022). PepSeA: Peptide Sequence Alignment and Visualization Tools to Enable Lead Optimization. J. Chem. Inf. Model..

[133] Li S., Wang X., Zhu Y., Ge J., Zhao D., Xu H., Hou T., Hsieh C.-Y. (2025). PepBAN: A Deep Learning Framework with Bilinear Attention and Adversarial Learning for Peptide–Protein Interaction Prediction. J. Chem. Inf. Model..

[134] Mao Q., Shang T., Xu W., Zhai S., Zhang C., Guo J., Su A., Li C., Duan H. (2025). NCPepFold: Accurate Prediction of Noncanonical Cyclic Peptide Structures via Cyclization Optimization with Multigranular Representation. J. Chem. Theory Comput..

[135] Xie X., Li C.Z., Lee J.S., Kim P.M. (2025). CyclicBoltz1, Fast and Accurately Predicting Structures of Cyclic Peptides and Complexes Containing Non-Canonical Amino Acids Using AlphaFold 3 Framework. bioRxiv.

[136] Zhu C., Cao S., Shang T., Guo J., Su A., Li C., Duan H. (2025). Predicting the Structures of Cyclic Peptides Containing Unnatural Amino Acids by HighFold2. Brief. Bioinform..

[137] Cao S., Zhu N., Duan H. (2025). Accurate Structure Prediction of Cyclic Peptides Containing Unnatural Amino Acids Using HighFold3. Brief. Bioinform..

[138] Wu D., Zou Y. (2026). Accurate 3D Structure Prediction of Small Cyclic Peptides Containing Non-Canonical Amino Acid Residues Using an All-Atom Diffusion Model with Stereogenic Implementation. J. Chem. Inf. Model..

[139] Zhong B., Zhang C., Guo S., Zhang C. (2017). Rational Design of Cyclic Peptides to Disrupt TGF-Β/SMAD7 Signaling in Heterotopic Ossification. J. Mol. Graph. Model..

[140] Fonseca Lopez F., Miao J., Damjanovic J., Bischof L., Braun M.B., Ling Y., Hartmann M.D., Lin Y.-S., Kritzer J.A. (2023). Computational Prediction of Cyclic Peptide Structural Ensembles and Application to the Design of Keap1 Binders. J. Chem. Inf. Model..

[141] Hosseinzadeh P., Watson P.R., Craven T.W., Li X., Rettie S., Pardo-Avila F., Bera A.K., Mulligan V.K., Lu P., Ford A.S. (2021). Anchor Extension: A Structure-Guided Approach to Design Cyclic Peptides Targeting Enzyme Active Sites. Nat. Commun..

[142] Mulligan V.K., Workman S., Sun T., Rettie S., Li X., Worrall L.J., Craven T.W., King D.T., Hosseinzadeh P., Watkins A.M. (2021). Computationally Designed Peptide Macrocycle Inhibitors of New Delhi Metallo-β-Lactamase 1. Proc. Natl. Acad. Sci. USA.

[143] Sun K., Li S., Zheng B., Zhu Y., Wang T., Liang M., Yao Y., Zhang K., Zhang J., Li H. (2024). Accurate de Novo Design of Heterochiral Protein-Protein Interactions. Cell Res..

[144] Soler M.A., Rodriguez A., Russo A., Adedeji A.F., Foumthuim C.J.D., Cantarutti C., Ambrosetti E., Casalis L., Corazza A., Scoles G. (2017). Computational Design of Cyclic Peptides for the Customized Oriented Immobilization of Globular Proteins. Phys. Chem. Chem. Phys..

[145] Delaunay M., Ha-Duong T. (2022). Des3PI: A Fragment-Based Approach to Design Cyclic Peptides Targeting Protein-Protein Interactions. J. Comput. Aided Mol. Des..

[146] Wang F., Zhang T., Zhu J., Zhang X., Zhang C., Lai L. (2025). Target-Based de Novo Design of Cyclic Peptide Binders. bioRxiv.

[147] Kosugi T., Ohue M. (2023). Design of Cyclic Peptides Targeting Protein–Protein Interactions Using AlphaFold. Int. J. Mol. Sci..

[148] Li Q., Vlachos E.N., Bryant P. (2024). Design of Linear and Cyclic Peptide Binders of Different Lengths Only from a Protein Target Sequence. bioRxiv.

[149] Rettie S.A., Juergens D., Adebomi V., Bueso Y.F., Zhao Q., Leveille A.N., Liu A., Bera A.K., Wilms J.A., Üffing A. (2025). Accurate de Novo Design of High-Affinity Protein Binding Macrocycles Using Deep Learning. Nat. Chem. Biol..

[150] Wu Z., Wu Y., Zhu C., Wu X., Zhai S., Wang X., Su Z., Duan H. (2023). Efficient Computational Framework for Target-Specific Active Peptide Discovery: A Case Study on IL-17C Targeting Cyclic Peptides. J. Chem. Inf. Model..

[151] Xu X., Xu C., He W., Wei L., Li H., Zhou J., Zhang R., Wang Y., Xiong Y., Gao X. (2024). HELM-GPT: De Novo Macrocyclic Peptide Design Using Generative Pre-Trained Transformer. Bioinformatics.

[152] Fang M., Wang C., Shi J., Lian F., Jin Q., Wang Z., Zhang Y., Chen P., Cui Z., Wang Y. (2026). HalluDesign: Protein Optimization and de Novo Design via Iterative Structure Hallucination and Sequence Design. bioRxiv.

[153] Cho Y., Rangel G., Bhardwaj G., Ovchinnikov S. (2025). Protein Hunter: Exploiting Structure Hallucination within Diffusion for Protein Design. bioRxiv.

[154] Zhai S., Liu T., Lin S., Li D., Liu H., Yao X., Hou T. (2025). Artificial Intelligence in Peptide-Based Drug Design. Drug Discov. Today.

[155] Zhang C., Xu Z., Lin K., Zhu N., Zhang C., Xu W., Guo J., Su A., Li C., Duan H. (2025). CycleDesigner: Leveraging CycRFdiffusion and HighFold to Design Cyclic Peptide Binders for Specific Targets. J. Chem. Inf. Model..

[156] Gimeno A., Ojeda-Montes M.J., Tomás-Hernández S., Cereto-Massagué A., Beltrán-Debón R., Mulero M., Pujadas G., Garcia-Vallvé S. (2019). The Light and Dark Sides of Virtual Screening: What Is There to Know?. Int. J. Mol. Sci..

[157] Leffler A.E., Kuryatov A., Zebroski H.A., Powell S.R., Filipenko P., Hussein A.K., Gorson J., Heizmann A., Lyskov S., Tsien R.W. (2017). Discovery of Peptide Ligands through Docking and Virtual Screening at Nicotinic Acetylcholine Receptor Homology Models. Proc. Natl. Acad. Sci. USA.

[158] Vincenzi M., Mercurio F.A., Leone M. (2024). Virtual Screening of Peptide Libraries: The Search for Peptide-Based Therapeutics Using Computational Tools. Int. J. Mol. Sci..

[159] Duffy F.J., O’Donovan D., Devocelle M., Moran N., O’Connell D.J., Shields D.C. (2015). Virtual Screening Using Combinatorial Cyclic Peptide Libraries Reveals Protein Interfaces Readily Targetable by Cyclic Peptides. J. Chem. Inf. Model..

[160] Chen Z., Li Y., Wang X., Qiu X., Wang C., Wang Z., Chen X., Wang J. (2024). A High-Throughput Molecular Dynamics Screening (HTMDS) Approach to the Design of Novel Cyclopeptide Inhibitors of ATAD2B Based on the Non-Canonical Combinatorial Library. J. Biomol. Struct. Dyn..

[161] Elokely K.M., Doerksen R.J. (2013). Docking Challenge: Protein Sampling and Molecular Docking Performance. J. Chem. Inf. Model..

[162] Zhai S., Tan Y., Zhu C., Zhang C., Gao Y., Mao Q., Zhang Y., Duan H., Yin Y. (2024). PepExplainer: An Explainable Deep Learning Model for Selection-Based Macrocyclic Peptide Bioactivity Prediction and Optimization. Eur. J. Med. Chem..

[163] Zhai S., Tan Y., Zhang C., Hipolito C.J., Song L., Zhu C., Zhang Y., Duan H., Yin Y. (2023). PepScaf: Harnessing Machine Learning with In Vitro Selection toward De Novo Macrocyclic Peptides against IL-17C/IL-17RE Interaction. J. Med. Chem..

[164] Roney J.P., Ovchinnikov S. (2022). State-of-the-Art Estimation of Protein Model Accuracy Using AlphaFold. Phys. Rev. Lett..

[165] Ding X., Chen X., Sullivan E.E., Shay T.F., Gradinaru V. (2024). Fast, Accurate Ranking of Engineered Proteins by Target-Binding Propensity Using Structure Modeling. Mol. Ther..

[166] Mondal A., Singh B., Felkner R.H., De Falco A., Swapna G., Montelione G.T., Roth M.J., Perez A. (2024). A Computational Pipeline for Accurate Prioritization of Protein-Protein Binding Candidates in High-Throughput Protein Libraries. Angew. Chem. Int. Ed. Engl..

[167] Lin Z., Akin H., Rao R., Hie B., Zhu Z., Lu W., Smetanin N., Verkuil R., Kabeli O., Shmueli Y. (2023). Evolutionary-Scale Prediction of Atomic-Level Protein Structure with a Language Model. Science.

[168] Zalewski M., Wallner B., Kmiecik S. (2025). Protein–Peptide Docking with ESMFold Language Model. J. Chem. Theory Comput..

[169] Li Z., Liu Z., Zhong J., Jiang Y. (2026). ProtT5-MSCRNet: A Multi-Scale Convolutional and Channel-Recalibrated Deep Learning Framework for Anticancer Peptide Prediction. Front. Artif. Intell..

[170] Waring M.J., Arrowsmith J., Leach A.R., Leeson P.D., Mandrell S., Owen R.M., Pairaudeau G., Pennie W.D., Pickett S.D., Wang J. (2015). An Analysis of the Attrition of Drug Candidates from Four Major Pharmaceutical Companies. Nat. Rev. Drug Discov..

[171] Nielsen D.S., Shepherd N.E., Xu W., Lucke A.J., Stoermer M.J., Fairlie D.P. (2017). Orally Absorbed Cyclic Peptides. Chem. Rev..

[172] Prentis R.A., Lis Y., Walker S.R. (1988). Pharmaceutical Innovation by the Seven UK-Owned Pharmaceutical Companies (1964–1985). Br. J. Clin. Pharmacol..

[173] Danhof M., De Lange E.C., Della Pasqua O.E., Ploeger B.A., Voskuyl R.A. (2008). Mechanism-Based Pharmacokinetic-Pharmacodynamic (PK-PD) Modeling in Translational Drug Research. Trends Pharmacol. Sci..

[174] Dougherty P.G., Wen J., Pan X., Koley A., Ren J.-G., Sahni A., Basu R., Salim H., Appiah Kubi G., Qian Z. (2019). Enhancing the Cell Permeability of Stapled Peptides with a Cyclic Cell-Penetrating Peptide. J. Med. Chem..

[175] Digiesi V., de la Oliva Roque V., Vallaro M., Caron G., Ermondi G. (2021). Permeability Prediction in the Beyond-Rule-of 5 Chemical Space: Focus on Cyclic Hexapeptides. Eur. J. Pharm. Biopharm..

[176] Rezai T., Bock J.E., Zhou M.V., Kalyanaraman C., Lokey R.S., Jacobson M.P. (2006). Conformational Flexibility, Internal Hydrogen Bonding, and Passive Membrane Permeability: Successful in Silico Prediction of the Relative Permeabilities of Cyclic Peptides. J. Am. Chem. Soc..

[177] Wang C.K., Northfield S.E., Swedberg J.E., Colless B., Chaousis S., Price D.A., Liras S., Craik D.J. (2015). Exploring Experimental and Computational Markers of Cyclic Peptides: Charting Islands of Permeability. Eur. J. Med. Chem..

[178] Frazee N., Billlings K.R., Mertz B. (2024). Gaussian Accelerated Molecular Dynamics Simulations Facilitate Prediction of the Permeability of Cyclic Peptides. PLoS ONE.

[179] Sugita M., Fujie T., Yanagisawa K., Ohue M., Akiyama Y. (2022). Lipid Composition Is Critical for Accurate Membrane Permeability Prediction of Cyclic Peptides by Molecular Dynamics Simulations. J. Chem. Inf. Model..

[180] Li J., Yanagisawa K., Sugita M., Fujie T., Ohue M., Akiyama Y. (2023). CycPeptMPDB: A Comprehensive Database of Membrane Permeability of Cyclic Peptides. J. Chem. Inf. Model..

[181] Cao L., Xu Z., Shang T., Zhang C., Wu X., Wu Y., Zhai S., Zhan Z., Duan H. (2024). Multi_CycGT: A Deep Learning-Based Multimodal Model for Predicting the Membrane Permeability of Cyclic Peptides. J. Med. Chem..

[182] Li J., Yanagisawa K., Akiyama Y. (2024). CycPeptMP: Enhancing Membrane Permeability Prediction of Cyclic Peptides with Multi-Level Molecular Features and Data Augmentation. Brief. Bioinform..

[183] Yu Y., Gu M., Guo H., Deng Y., Chen D., Wang J., Wang C., Liu X., Yan W., Huang J. (2024). MuCoCP: A Priori Chemical Knowledge-Based Multimodal Contrastive Learning Pre-Trained Neural Network for the Prediction of Cyclic Peptide Membrane Penetration Ability. Bioinformatics.

[184] Baker C., Acquah F.A., Chivukula L.G., Wu L., Philippe-Venec L., Abedi M., Tao Y., Ramírez D., McCoy M.D., Moore B. (2026). PEGASUS: Unlocking Polarity in Cell-Permeable Cyclic Peptides Using AI Models Built on Massively Parallel Biological Assays. J. Med. Chem..

[185] Liu W., Li J., Verma C.S., Lee H.K. (2025). Systematic Benchmarking of 13 AI Methods for Predicting Cyclic Peptide Membrane Permeability. J. Cheminform.

[186] Krishna R., Wang J., Ahern W., Sturmfels P., Venkatesh P., Kalvet I., Lee G.R., Morey-Burrows F.S., Anishchenko I., Humphreys I.R. (2024). Generalized Biomolecular Modeling and Design with RoseTTAFold All-Atom. Science.

[187] Feller A.L., Wilke C.O. (2025). Peptide-Aware Chemical Language Model Successfully Predicts Membrane Diffusion of Cyclic Peptides. J. Chem. Inf. Model..

[188] Tobias J.W., Shrader T.E., Rocap G., Varshavsky A. (1991). The N-End Rule in Bacteria. Science.

[189] Varshavsky A. (1997). The N-End Rule Pathway of Protein Degradation. Genes Cells.

[190] Mathur D., Singh S., Mehta A., Agrawal P., Raghava G.P.S. (2018). In Silico Approaches for Predicting the Half-Life of Natural and Modified Peptides in Blood. PLoS ONE.

[191] Cavaco M., Valle J., Flores I., Andreu D., A R B Castanho M. (2021). Estimating Peptide Half-Life in Serum from Tunable, Sequence-Related Physicochemical Properties. Clin. Transl. Sci..

[192] Tan X., Liu Q., Fang Y., Yang S., Chen F., Wang J., Ouyang D., Dong J., Zeng W. (2024). Introducing Enzymatic Cleavage Features and Transfer Learning Realizes Accurate Peptide Half-Life Prediction across Species and Organs. Brief. Bioinform..

[193] Yu Q., Zhang Z., Liu G., Li W., Tang Y. (2024). ToxGIN: An In Silico Prediction Model for Peptide Toxicity via Graph Isomorphism Networks Integrating Peptide Sequence and Structure Information. Brief. Bioinform..

[194] Ebrahimikondori H., Sutherland D., Yanai A., Richter A., Salehi A., Li C., Coombe L., Kotkoff M., Warren R.L., Birol I. (2024). Structure-Aware Deep Learning Model for Peptide Toxicity Prediction. Protein Sci..

[195] Hu H., Zhang C., Xu Z., Guo J., Su A., Li C., Duan H. (2025). PepMSND: Integrating Multi-Level Feature Engineering and Comprehensive Databases to Enhance in vitro/in vivo Peptide Blood Stability Prediction. Digit. Discov..

[196] Cifuentes P., Adàlia R., Zamora I. (2025). Prediction of Peptide Cleavage Sites Using Protein Language Models and Graph Neural Networks. Sci. Rep..

[197] Ohta A., Tanada M., Shinohara S., Morita Y., Nakano K., Yamagishi Y., Takano R., Kariyuki S., Iida T., Matsuo A. (2023). Validation of a New Methodology to Create Oral Drugs beyond the Rule of 5 for Intracellular Tough Targets. J. Am. Chem. Soc..

[198] Halbwedl N., Zacharias M. (2026). AlphaFold2-Guided Cyclic Peptide Stabilizer Design to Target Protein–Protein Interactions. Proteins Struct. Funct. Bioinform..

